# Analytical model enabling the correction of absorption and scattering in dual-color ratiometric multiphoton microscopy of brain tissue

**DOI:** 10.1117/1.NPh.13.3.035007

**Published:** 2026-09-03

**Authors:** Emmanuelle Chaigneau, Marine Tournissac, Sophie Walter, Mayeul Collot, Serge Charpak

**Affiliations:** a Sorbonne Université, INSERM, CNRS, Institut de la Vision, Paris, France; b Université de Strasbourg, CNRS, Laboratoire de Chémo-Biologie Synthétique et Thérapeutique (CBST) UMR 7199, Chemistry of Photoresponsive Systems, Illkirch, France

**Keywords:** multiphoton microscopy, ratiometric measurements, cerebral cortex imaging, absorption, scattering

## Abstract

**Significance:**

Multiphoton microscopy with fluorescent indicators enables the monitoring of biomarker dynamics through changes in fluorescence intensity. However, quantitative interpretation remains challenging because fluorescence signals are highly sensitive to experimental conditions. Dual-color ratiometric approaches improve quantification, yet wavelength-dependent absorption and scattering of emitted photons can still introduce significant bias.

**Aim:**

This study aimed to develop a correction method for absorption and scattering effects to achieve unbiased dual-color ratiometric measurements in multiphoton microscopy.

**Approach:**

We developed an analytical model that incorporates the spectral optical properties of biological tissues and fluorophores. The model was applied to the rodent cerebral cortex parenchyma and to the lumen of individual blood vessels. Its predictions were evaluated using simulations and *in vivo* two-photon microscopy experiments.

**Results:**

In the cerebral cortex parenchyma, absorption and scattering bias dual-color ratiometric measurements by up to ∼100% at depths of approximately ∼400  μm. Our model accurately describes and corrects these effects. Within the vessel lumen, however, the situation is more complex: the model effectively corrects the biases in veins, but not in arterioles.

**Conclusions:**

Overall, the proposed model enhances the accuracy of biomarker quantification in multiphoton microscopy and provides practical tools for correcting optical biases in biological tissues.

## 1 Introduction

Multiphoton microscopy combined with fluorescent sensors are widely used to report changes of biomarkers in their environment, such as calcium ions,^1^^–^^3^ protons,^4^ metabolites,^5^ or neurotransmitters. Traditionally, time-dependent variations in these biomarkers are quantified by normalizing the fluorescence signal of the reporter at time t (F(t)) to a baseline fluorescence ($F_{0}$) calculated as the average of F(t) over a selected time window after background subtraction ($F_{b}$). However, commonly used fluorescence ratios as $\frac{F(t)-F_{b}}{F_{0}-F_{b}}$ or $\frac{F(t)-F_{0}}{F_{0}-F_{b}}$ are not strictly quantitative as they depend on multiple experimental and optical parameters. First, the relationship between fluorescence and biomarker activity depends on the affinity of the reporter, which must be appropriate to capture the full dynamic range of the biomarker signal in the medium of interest. In recent years, an expanding repertoire of chemical sensors and genetically encoded biosensors has been developed,^5^^–^^7^ enabling increasingly quantitative measurements of a wide range of biomarkers. Second, fluorescence ratios are strongly influenced by the optical properties of biological tissues (reviewed by Ref. 8), which affect both the excitation beam and fluorescence emission.^9^ These optical properties can vary over time and with tissue motion, leading to time-dependent fluctuations in measured fluorescence signals. Hemoglobin, in particular, is a major absorber for both excitation and emitted photons, owing to its high one- and two-photon absorption cross-sections and their dependence on oxygenation state.^10^^,^^11^ Consequently, dynamic changes in blood volume or oxygenation along the photon path can substantially bias multiphoton measurements. Similar optical artefacts have been reported in other imaging modalities, such as wide-field optical imaging (reviewed by Ref. 12) or fiber photometry,^13^ prompting the development of various correction strategies.^12^^–^^15^

**Fig. 1:**
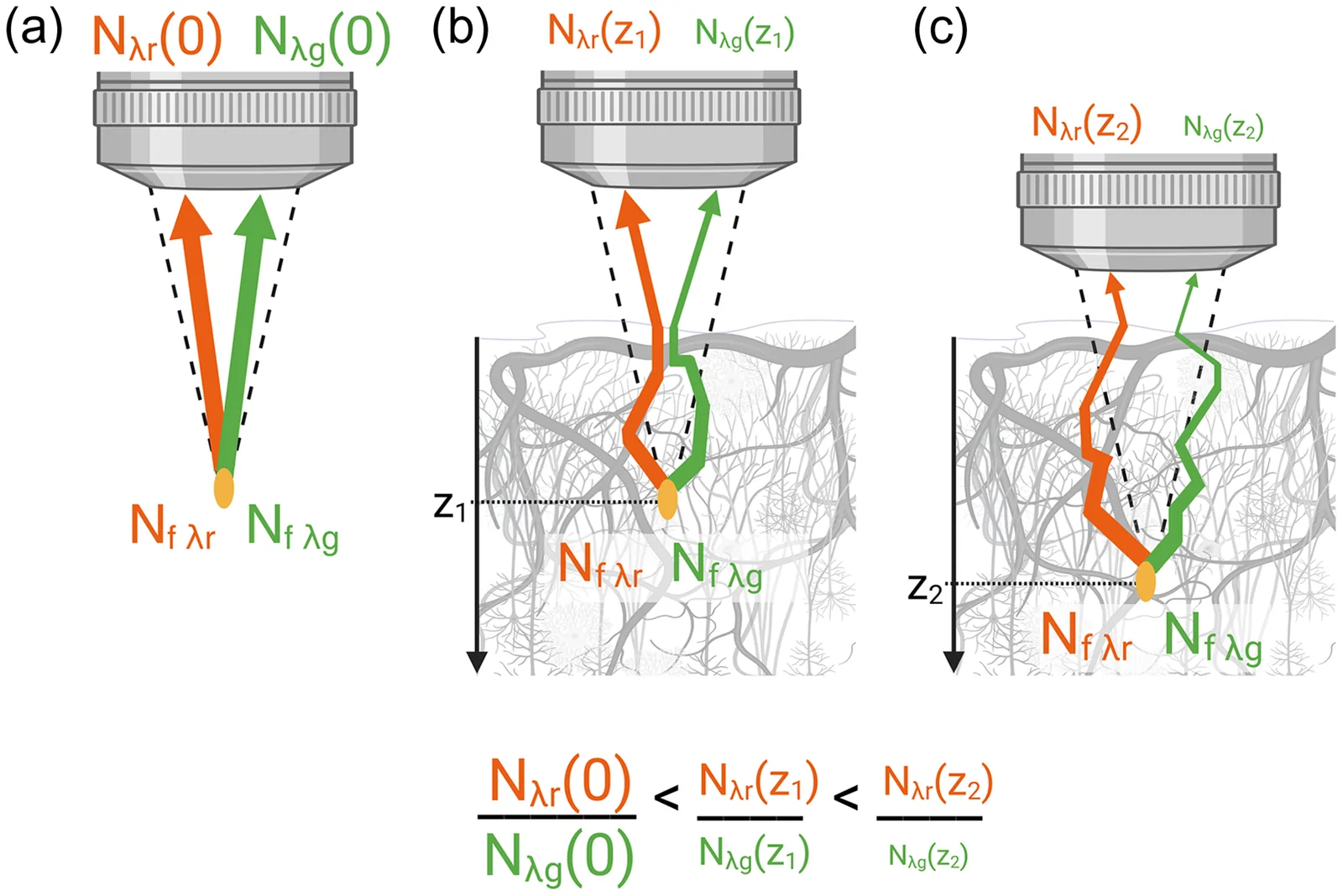
Schematic illustrating the effect of absorption and scattering on the ratio of the number of photons collected at two wavelengths (*e.g.*, red and green). The excitation beam (dashed lines) simultaneously excites red- and green-emitting fluorophores. The excitation power is adjusted so that the same number of fluorescence photons is generated at the focal point (yellow spot), independent of the sample conditions: $N_{f}\lambda_{r}$ for red photons and $N_{f}\lambda_{g}$ for green photons. (a) In a transparent sample, the collected photons ($N\lambda_{r}$ for red and $N\lambda_{g}$ for green) correspond to those emitted within the excitation cone. The ratio of collected photons is: $\frac{N_{\lambda r}(0)}{N_{\lambda g}(0)}=\frac{\alpha_{f\lambda r}N_{f\lambda r}}{\alpha_{f\lambda g}N_{f\lambda g}}$ where $\alpha_{f}\lambda_{r}$ and $\alpha_{f}\lambda_{g}$ are the respective collection efficiencies of the system for green and red photons. This ratio, $\frac{N_{\lambda r}(0)}{N_{\lambda g}(0)}$ is also the ratio measured at the surface of any sample. Panels (b)–(c) show when the focal point is located within tissue, emitted photons undergo absorption and scattering by cells and blood vessels (in grey). As these effects are stronger for green photons than for red photons, the ratio of the collected red to green photons increases with imaging depth.

In multiphoton microscopy, the shape, size, and intensity of the excitation beam are affected by wavefront distortion, as well as by scattering and absorption within living tissue.^16^^–^^18^ In addition, the number of collected fluorescence photons decreases with imaging depth^19^ and depends on wavelength,^20^ owing to absorption and scattering. Correcting for these effects is particularly challenging because of their high spatial variability.^17^ To overcome these limitations, imaging strategies that are independent of the absolute number of collected fluorescence photons have been developed, including fluorescence lifetime and ratiometric measurements. Fluorescence lifetime approaches rely on probes whose excited-state lifetime changes upon biomarker binding or in response to environmental variations such as pH or viscosity.^21^^,^^22^ In this context, the mean fluorescence lifetime serves as a quantitative reporter of the biomarker concentration, independent of other factors. Alternatively, dual-color ratiometric methods exploit either the spectral properties of a single sensor, whose excitation or emission spectrum varies with biomarker concentration,^23^^,^^24^ or the combination of an active sensor with a spectrally distinct, inert reference fluorophore.^25^ By taking the ratio of two fluorescent signals, these approaches substantially reduce the effect of extrinsic factors on the measurement. In multiphoton microscopy, the most common dual-color ratiometric strategy uses fluorophores that can be excited at a single wavelength but emit in sufficiently separated spectral bands, allowing their contributions to be distinguished using dual-channel detection. Computing the ratio of the two emission signals compensates for variations in excitation efficiency and sample motion. In optically transparent and nonabsorbing media, this approach enables accurate biomarker quantification with simple calibration procedures. However, in biological tissue, wavelength-dependent absorption and scattering of emitted photons distort the fluorescence ratio, an effect that becomes increasingly pronounced with imaging depth^20^^,^^26^ (Fig. 1).

Here, we developed a mathematical model for dual-color ratiometric measurements that incorporates the effects of absorption and scattering of the tissue on emitted light, enabling unbiased biomarker quantification. We tested this model using *in vivo* two-photon microscopy imaging in the mouse brain vasculature. Our goal is to provide a practical tool to assess and correct the impact of optical parameters on dual-color ratiometric measurements with epifluorescence collection, thereby enabling accurate biomarker quantification in biological tissues.

## 2 Materials and Methods

### 2.1 Mathematical Model and Simulations

We developed an analytical mathematical model based on multiphoton optics in absorbing and scattering samples to describe ratiometric measurements (Sec. 3.1). This model was then implemented in MATLAB to perform numerical simulations under conditions representative of two-photon imaging in the cerebral cortex (Secs. 3.2 and 3.3). In the first set of simulations, we considered two neutral fluorophores: Texas Red, a sulforhodamine 101 derivative emitting in the red spectral range, and fluorescein, which emits in the green spectral range. Fluorescence detection was simulated in the green spectral band from 500 to 550 nm and in the red spectral band from 604 to 644 nm. The second set of simulations focused on the measurement of a biomarker: blood pH. We used the red-emitting pH sensor H-Ruby^4^^,^^27^^,^^28^ in combination with the pH-insensitive green-emitting fluorophore Alexa Fluor 488. Fluorescence collection was simulated in the green spectral band from 500 to 550 nm and in the red spectral band from 578 to 640 nm.

### 2.2 Dual Color Ratiometric Multiphoton Image Acquisition

*In vivo* experiments were performed to validate the mathematical model (Sec. 3.3), using identical fluorophores and spectral collection bands to those used in numerical simulations. Two-photon (2P) imaging was performed in the cerebral cortex of sedated mice implanted with a chronic cranial window (Sec. 2.4 for description of animal procedures), using a custom-built microscope. A femtosecond laser (Mai Tai eHP; Spectra-Physics) with a dispersion compensation module (Deepsee; Spectra-Physics) emitting 70-fs pulses at 80 MHz was used as a light source. The laser power was attenuated by an acoustic optical modulator (AA Optoelectronic, MT110-B50-A1.5-IR-Hk). Scanning was performed with galvanometric scanner mirrors (8315KM60B; Cambridge Technology) and focused into the samples with a water immersion objective, either a LUMFLN60XW (Olympus, 1.1 NA) or a LUMPLFLN40XW (Olympus, 0.8 NA), conjugated to the scanning system by relay optics. Epifluorescence was collected with the same objective, separated from excitation light with a dichroic mirror centered at 775 nm, and sorted according to their wavelength using a dichroic mirror centered at 570 nm and a FF01-525/50 nm filter (Semrock) for AF488 and fluorescein, a FF01-609/62 (Semrock) for H-Ruby, and a FF01-624/40 (Semrock) for Texas Red. Photons were collected with two GaAsP photomultipliers tubes (H10770A-40 from Hamamatsu), and the output signal was converted using transimpedances (C7319 from Hamamatsu). Custom-made electronics and LabView software were used to control the system.

In the first series of experiments, 70 kDa Texas Red Dextran (Invitrogen D1830) and 70 kDa fluorescein Dextran (Invitrogen D1823), with respective degrees of labelling of 4 for Texas Red and 6 to 7 for fluorescein, were injected into the bloodstream. The fluorophores reached blood concentrations of $1.43\times 10^{-5}\;M$ for Texas Red and 2.14 to $2.5\times 10^{-5}\;M$ for fluorescein and were imaged at an excitation wavelength of 920 nm. At this wavelength, the 2P action cross section (defined as the product of the 2P cross-section and the quantum yield) of Texas Red (Sulforhodamine 101 acid chloride) and fluorescein are respectively ∼110 GM ^29^ and ∼18 GM (average of Refs. 29–31), respectively.

In the second series of experiments, which focused on pH measurements, either 70 kDa H-Ruby-Ethyl dextran^27^ or 70 kDa H-Ruby-PEG dextran ^4^ with respective degrees of labelling of 0.5 to 2.4, together with 70 kDa AF488 dextran with respective degrees of labelling of 0.7 to 4.2 (all provided by M. Collot laboratory), were injected in the bloodstream. The resulting blood concentrations ranged from $2.6\times 10^{-6}$ to $4.8\times 10^{-6}\;M$ for the H-Ruby derivatives, and from $5\times 10^{-6}$ to $2.4\times 10^{-5}\;M$ for AF488. Imaging was performed using an excitation wavelength of 880 nm. At this wavelength, the 2P action cross-sections of H-Ruby-Et and H-Ruby-PEG were 26 and 122 GM, respectively,^4^ whereas the 2P action cross-section of AF488 was measured and found to be 11 GM, close to the average of Refs. 32 and 33.

Standard raster scans were acquired at 160.00  kHz/pixel. Image averaging was applied to maximize the signal-to-noise ratio.

The area through which collected photons exit the tissue is at least as large as the intersection of the excitation cone with the brain surface. This intersection depends on the imaging depth and the numerical aperture of the objective. For instance, imaging at a depth of 200  μm with a 1.1 NA objective corresponds to a cone measuring 200  μm in height and 250  μm in radius at the surface. Only images obtained from regions where the excitation cone was fully contained within brain tissue were included in the analysis; regions partially covered by bone or dental cement were excluded.

### 2.3 Dual-Color Ratiometric Multiphoton Image Analysis in Vessels

Images were analyzed using a custom-made software developed with LabView. For each region of interest (ROI) within a blood vessel, the ratio of the mean red to the mean green fluorescence signals was computed after subtraction of their respective background signals. This ratio was measured at discrete depths ($z_{i}$) and discrete time points ($t_{i}$) following fluorophores injection and is defined as: $\text{FRatio}(z_{i},t_{i})=\frac{F_{\text{Red}}(z_{i},t_{i})-F_{\text{RedBackground}}(z_{i},t_{i})}{F_{\text{Green}}(z_{i},t_{i})-F_{\text{GreenBackground}}(z_{i},t_{i})}$. Because the plasmatic concentration of each fluorophore decreases over time at fluorophore- and animal-specific rate, the measured fluorescence in each spectral band depends on both depth and time. To isolate depth-dependent effects, $\mathrm{FRatio}(z_{i},t_{i})$ needs to be normalized by the fluorescence ratio at the surface, and at the same time, FRatio (0, $t_{i}$) (Fig. 11 in Appendix A). Because measurements at depth and at the surface were not acquired simultaneously, we first modeled the time-dependence of changes in the concentration of fluorophores (TDCCF) at the surface to estimate the fluorescence ratio at the surface FRatio M(0,t) at any time (Fig. 11 in Appendix A). The surface ratio was fitted with an exponential function: $\mathrm{FRatio}\;M(0,t)=a+be^{t/\tau }$, where a, b and τ are constants determined from experimental surface data. Depth-dependent ratios were then corrected for TDCCF by computing $\frac{\text{FRatio}(z,t)}{\mathrm{FRatio}\;M(0,t)}$ (Fig. 11 in Appendix A). The final normalized depth-dependent value $\frac{\text{FRatio}(z)}{\mathrm{FRatio}\;M(0)}$ was obtained by averaging $\frac{\text{FRatio}(z,t)}{\mathrm{FRatio}\;M(0,t)}$ over all time points. Depth-dependent fitting was performed with MATLAB using a least-square optimization procedure.

**Fig. 2:**
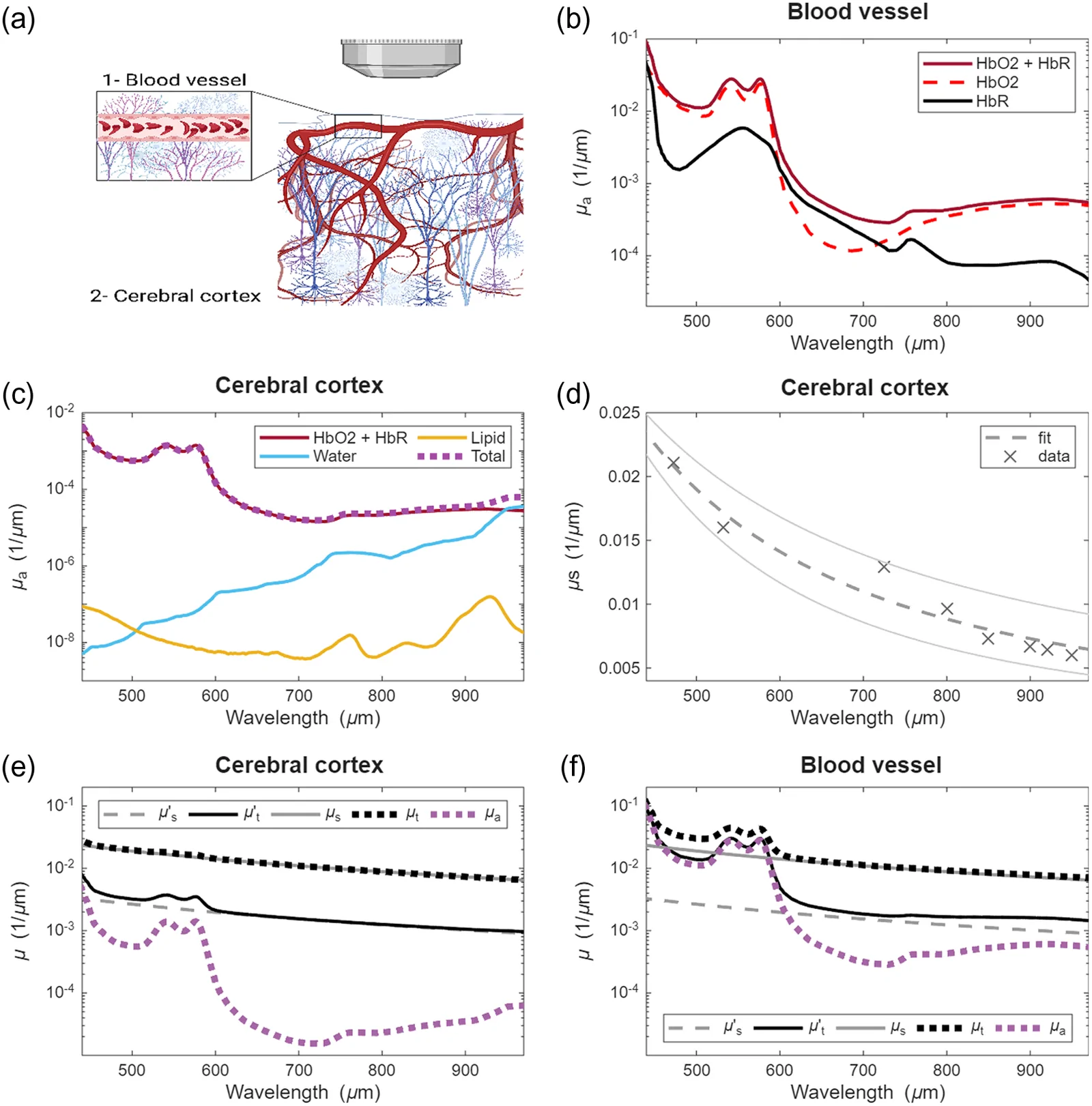
Wavelength dependence of absorption and scattering in the cerebral cortex. (a) Schematic of the mouse cerebral cortex. (b) Absorption spectrum within a blood vessel, showing the respective contributions of oxy- hemoglobin (${\mathrm{HbO}}_{2}$) and deoxy-hemoglobin (HbR) weighted by their relative proportions. (c) Absorption spectrum of the cerebral cortex and its individual components, weighted by their relative proportions, assuming a blood volume fraction $\chi_{\mathrm{Blood}\;\mathrm{CC}}=5\%$. (d) Modelling of the cerebral cortex scattering coefficient $\mu_{s}$ based on experimental data from^17^^,^^57^^,^^60^^,^^61^ (Table 1). (e) Wavelength-dependence of absorption ($\mu_{a}$), scattering ($\mu_{s}$), total attenuation ($\mu_{t}$), reduced scattering ($\mu_{s}^{\prime }$) and reduced total attenuation ($\mu_{t}^{\prime }$) coefficients in the cerebral cortex. (f) Same as (e) in a blood vessel.

### 2.4 Animal Care and Experimentation

All animal care and experimentations were performed in accordance with the INSERM Animal Care and Use Committee guidelines and approved by the ethical committee Charles Darwin (protocol number #27135 2020091012114621). Mice were fed ad libitum and housed in a 12-h light-dark cycle. Eight Adult C57/BL6J mice, both males and females, from 3 to 12 months of age were included. Chronic cranial windows with 100  μm-thick glass coverslips over the barrel cortex were implanted one week after the head bar surgery as described in Ref. 34. A minimum recovery period of 7 to 10 days was respected before imaging experiments. During imaging sessions, mice were either anesthetized with a mixture of ketamine and medetomidine (100 and 0.5  mg/kg, respectively, intraperitoneal) or sedated with a continuous perfusion of dexmedetomidine (induction with 3% isoflurane, which was decreased by 0.5% every 5 min down to 0% within 30 min, subcutaneous bolus of dexmedetomidine 0.025  mg/kg at the beginning, and subcutaneous perfusion of 0.1  mg/kg/h dexmedetomidine during the entire session). Dyes were administered intravenously with a retro-orbital injection prior to the decrease in isoflurane (under 3% isoflurane) or under ketamine/medetomidine anesthesia. Recordings started 20 min after the isoflurane cutoff. Mice received air continuously through a nose cone supplemented with oxygen to reach a final concentration of 28% oxygen. Mice’s body temperature was controlled with a rectal probe and maintained at 36.5°C with a feedback-controlled heating pad. The animal was monitored throughout the imaging session using an infrared webcam (DCC3240N, Thorlabs). Breathing was monitored using a thermocouple placed in front of one nostril.^35^

## 3 Results

### 3.1 Development of the Mathematical Model

We considered two fluorophores, A and B, located within the same focal volume, excited simultaneously by multiphoton excitation (Fig. 1) and epi-fluorescence collection by broad detectors in spectral bands [λ1;λ2] and [λ3;λ4], where λ1<λ2<λ3<λ4. Here, we assume that A emits mostly in the [λ3;λ4] band and does not emit fluorescent photons at wavelengths below λ2. We also assume that B emits mostly in the band [λ1;λ2], but that its emission in the spectral band [λ3;λ4] cannot be neglected. The opposite case can be solved by simply inverting A and B in the following equations.

#### 3.1.1 Photons emitted at the focal point and collected in a transparent sample

The average fluorescence photon flux emitted at the focal point at wavelength λ by fluorophores A and B in response to n photons (nP) excitation can be estimated by^30^^,^^36^:

$$N_{0A}(\lambda ,[A])=\frac{[A]\sigma_{nPA}\phi_{A}\varphi_{NA}(\lambda )g^{\left(n\right)}I^{n}V_{n\mathrm{PPSF}}}{n},$$ (1)

$$N_{0B}(\lambda ,[B])=\frac{[B]\sigma_{nPB}\phi_{B}\varphi_{NB}(\lambda )g^{\left(n\right)}I^{n}V_{\mathrm{nP}\mathrm{PSF}}}{n}.$$ (2)

[A] and [B] denote the respective concentrations of fluorophores A and B. The parameters $\sigma_{nPA}$ and $\sigma_{nPB}$ are the corresponding nP cross-sections of A and B. The parameters $\phi_{A}$ and $\phi_{B}$ are the respective standard fluorescence quantum yields of A and B, defined as the ratio of the radiative decay rate to the total decay rate of the excited singlet state. The functions $\varphi_{NA}(\lambda )$ and $\varphi_{NB}(\lambda )$ represent the normalized differential quantum efficiencies of fluorescence of A and B, respectively, and satisfy the normalization conditions, $\int_{0}^{\infty }\varphi_{NA}(\lambda )d\lambda =1$ and $\int_{0}^{\infty }\varphi_{NB}(\lambda )d\lambda =1$. The term $g^{\left(n\right)}$ is the nth order temporal coherence of the excitation source. I is the time- and space-averaged intensity of excitation photons within the nP point spread function (nP PSF), with an effective volume is $V_{\mathrm{nP}\mathrm{PSF}}$.

In a transparent sample, the time-averaged number of photons that will be collected by the detectors with an optical transmission α(λ) in spectral ranges [λ1;λ2] and [λ3;λ4] can be estimated as:

$$N_{\lambda 1\lambda 2}([B],z=0)=\frac{g^{\left(n\right)}I^{n}V_{\mathrm{nPPSF}}}{n}[B]\sigma_{nPB}\phi_{B}\Phi_{N0B12},$$ (3)

$$N_{\lambda 3\;\lambda 4}([A],[B],z=0)=\frac{g^{\left(n\right)}I^{n}V_{\mathrm{nPPSF}}}{n}([A]\sigma_{nPA}\phi_{A}\Phi_{N0A34}+[B]\sigma_{nPB}\phi_{B}\Phi_{N0B34}),$$ (4)

where the constants $\Phi_{N0B12}$, $\Phi_{N0B34}$ and $\Phi_{N0A34}$ represent the quantum efficiencies scaled by optical transmission of B and A restricted to [λ1;λ2] and [λ3;λ4]:

$$\Phi_{N0\;B12}=\int_{\lambda 1}^{\lambda 2}\alpha (\lambda )\varphi_{NB}(\lambda )d\lambda ,$$ (5)

$$\Phi_{N0\;B34}=\int_{\lambda 3}^{\lambda 4}\alpha (\lambda )\varphi_{NB}(\lambda )d\lambda ,$$ (6)

$$\Phi_{N0\;A34}=\int_{\lambda 3}^{\lambda 4}\alpha (\lambda )\varphi_{NA}(\lambda )d\lambda .$$ (7)

Their ratio can be estimated by:

$$\frac{N_{\lambda 3\;\lambda 4}}{N_{\lambda 1\;\lambda 2}}([A],[B],z=0)=\frac{[A]\sigma_{N}}{\left[B\right]}\frac{\Phi_{N0A34}}{\Phi_{N0B12}}+\frac{\Phi_{N0B34}}{\Phi_{N0B12}},$$ (8)

$$\sigma_{N}=\frac{\sigma_{nPA}\phi_{A}}{\sigma_{nPB}\phi_{B}}.$$ (9)

Therefore, in a transparent sample, the ratio of photons collected in the two spectral bands is independent from the excitation beam. At the focal point it depends only on the optical transmission of the system, on the fluorophore concentrations and on their photochemical properties.

#### 3.1.2 Propagation in the tissue

We then considered interactions between tissue and emitted photons, i.e., absorption and scattering, and their impact on the ratio of detected photons. To build our model, we assumed that the sample is sufficiently homogeneous such that its optical properties remain constant throughout the volume. Consequently, the absorption and scattering coefficients, $\mu_{a}(\lambda )$ and $\mu_{s}(\lambda )$, and the anisotropy of scatter $g_{s}(\lambda )=\langle \cos\;\theta \rangle (\lambda )$, where θ is the scattering angle and which quantifies the direction of scatter,^8^^,^^37^^,^^38^ depend only on wavelength.

Consequently, the total attenuation coefficient $\mu_{t}(\lambda )=\mu_{a}(\lambda )+\mu_{s}(\lambda )$, which characterizes the overall loss of photons along a given direction due to absorption and scattering, and the reduced total attenuation coefficient, $\mu_{t}^{\prime }(\lambda )=\mu_{a}(\lambda )+(1-g_{s})\mu_{s}(\lambda )$, which characterizes photon loss due to absorption or scattering events that deviate photons from the forward direction, both depend solely on wavelength.

In multiphoton microscopy, fluorescence emission is isotropic and, under standard experimental conditions, occurs exclusively from the focal point.^39^^,^^40^ We therefore assumed that the photons were emitted from a point source located at depth z along the optical axis, and that collection of the emitted photons with broad detectors could be modelled as a function of z.

We first investigated the effect of absorption alone. When absorption is the only interaction considered, the number of photons of wavelength λ reaching the detector can then be modeled as an exponential decay with depth in the tissue, governed by the sample’s absorption coefficient, which acts as the extinction coefficient.^41^ This leads to an absorption-only (Ab) model, in which the ratio of photons collected in the two spectral bands is given by:

$${\left(\frac{N_{\lambda 3\;\lambda 4}}{N_{\lambda 1\;\lambda 2}}\right)}_{\mathrm{Ab}}([A],[B],z)=\frac{[A]\sigma_{N}}{\left[B\right]}\frac{{\left(\Phi_{N\;A34}\left(z\right)\right)}_{\mathrm{Ab}}}{{\left(\Phi_{N\;B12}(z)\right)}_{\mathrm{Ab}}}+\frac{{\left(\Phi_{N\;B34}(z)\right)}_{\mathrm{Ab}}}{{\left(\Phi_{N\;B12}(z)\right)}_{\mathrm{Ab}}},$$ (10)

$${\left(\Phi_{N\;A34}(z)\right)}_{\mathrm{Ab}}=\int_{\lambda 3}^{\lambda 4}\alpha (\lambda )\varphi_{NA}(\lambda )e^{-\mu_{a}(\lambda )z}d\lambda ,$$ (11)

$${\left(\Phi_{N\;B34}(z)\right)}_{\mathrm{Ab}}=\int_{\lambda 3}^{\lambda 4}\alpha (\lambda )\varphi_{N\;B}(\lambda )e^{-\mu_{a}\left(\lambda \right)z}d\lambda ,$$ (12)

$${\left(\Phi_{N\;B12}(z)\right)}_{\mathrm{Ab}}=\int_{\lambda 1}^{\lambda 2}\alpha (\lambda )\varphi_{N\;B}(\lambda )e^{-\mu_{a}(\lambda )z}d\lambda .$$ (13)

We then considered the combined effects of absorption and scattering. In this case, determining the exact number of photons reaching the detector requires solving the radiative transfer equation (RTE), which does not admit a closed-form solution. As a result, several diffusion approximations (DAs) have been proposed (early developments reviewed by Ref. 42, followed by Refs. 43–45). Conventional DAs model photon attenuation as a depth-dependent exponential decay governed by an effective extinction coefficient, assumed to be either $\mu_{t}^{\prime }(\lambda )$, $\mu_{t}(\lambda )$ or an intermediate value obtained through a fixed correction factor.

In a preliminary version of this project, we adopted such a depth-dependent exponential attenuation with wavelength-dependent extinction coefficients,^46^ which provided a first estimate of depth-dependent change in the ratio of collected photons. However, DAs using an exponential decay with a constant effective extinction coefficient are valid only under restricted conditions: when absorption is weak compared with scattering, when the anisotropy of scatter is limited, and at depths large in comparison to the scattering length ($1/\mu_{s}(\lambda )$).^47^ These conditions are generally not satisfied for emitted light in 2-photon imaging of biological tissues, where scattering anisotropy is high and imaging depths are in the same order of magnitude as the scattering length.

To overcome these limitations, Tricoli et al. ^48^ developed a new DA that remains valid at arbitrary depths. Rather than relying on an effective extinction coefficient that is constrained by a fixed relationship with $\mu_{a}(\lambda )$, $\mu_{t}(\lambda )$ and $g_{s}(\lambda )$, they introduce a reduced extinction coefficient $\mu_{e}(\lambda )$, whose relationship to these optical properties is allowed to vary when solving the DA of the RTE. This additional degree of freedom enables more accurate representation of both the angular distribution of the scattering anisotropy and the relative magnitude of the absorption-to-scattering coefficient ratio within the medium. As a result, the DA remains accurate even at imaging depths comparable to the scattering mean free path. In the resulting solution, $\mu_{e}(\lambda )$ defines one of the two depth-dependent attenuation terms, while the second is described by the conventional attenuation coefficient $k_{d}(\lambda )=\sqrt{3\mu_{a}(\lambda )\mu_{t}^{\prime }(\lambda )}$. Consequently, the number of photons at depth z is proportional to:

$$F(\lambda )e^{-\mu_{e}(\lambda )z}+G(\lambda )e^{-k_{d}(\lambda )z},$$ (14)

with,

$$F(\lambda )=\frac{-2\mu_{e}(\lambda {)}^{2}}{\mu_{e}(\lambda {)}^{2}-k_{d}(\lambda {)}^{2}},$$ (15)

$$G(\lambda )=\frac{3\mu_{e}(\lambda {)}^{2}-k_{d}(\lambda {)}^{2}}{\mu_{e}(\lambda {)}^{2}-k_{d}(\lambda {)}^{2}}+\frac{3l(\lambda )}{1+k_{d}(\lambda )l(\lambda )}(\mu_{t}^{\prime }\left(\lambda \right)-\frac{\mu_{e}(\lambda )k_{d}(\lambda )+k_{d}(\lambda {)}^{2}/3}{\mu_{e}(\lambda )+k_{d}(\lambda )}),$$ (16)

where l(λ) is the extrapolated boundary distance, inversely proportional to $\mu_{t}^{\prime }(\lambda )$: $l(\lambda )=\frac{\alpha_{L}}{\mu_{t}^{\prime }(\lambda )}$.

In the regime where absorption is negligible compared to scattering, this model converges toward a depth-dependent exponential decay. Indeed, because $\mu_{e}(\lambda )$ lies between $\mu_{t}^{\prime }(\lambda )$ and $\mu_{t}(\lambda )$, the condition of negligible absorption implies, $\sqrt{3\mu_{a}(\lambda )}\ll \sqrt{\mu_{t}^{\prime }(\lambda )}$. As a result, $e^{-\mu_{e}(\lambda )z}\ll e^{-k_{d}(\lambda )z}$. Consequently, at large depths, the photon density can be approximated as $N(\lambda ,z)\approx G(\lambda )e^{-k_{d}(\lambda )z}$.

Using Eq. (14), the ratio of photons collected in the two spectral bands in the presence of absorption and scattering (AbSc) can be estimated by:

$${\left(\frac{N_{\lambda 3\;\lambda 4}}{N_{\lambda 1\;\lambda 2}}\right)}_{\mathrm{AbSc}}([A],[B],z)=\frac{[A]\sigma_{N}}{\left[B\right]}\frac{{\left(\Phi_{N\;A34}\left(z\right)\right)}_{\mathrm{AbSc}}}{{\left(\Phi_{N\;B12}(z)\right)}_{\mathrm{AbSc}}}+\frac{{\left(\Phi_{N\;B34}(z)\right)}_{\mathrm{AbSc}}}{{\left(\Phi_{N\;B12}(z)\right)}_{\mathrm{AbSc}}},$$ (17)

$${\left(\Phi_{N\;A34}(z)\right)}_{\mathrm{AbSc}}=\beta_{A34}\int_{\lambda 3}^{\lambda 4}\alpha (\lambda )\varphi_{N\;A}(\lambda )(F(\lambda )e^{-\mu_{e}(\lambda )z}+G(\lambda )e^{-k_{d}(\lambda )z})d\lambda ,$$ (18)

$${\left(\Phi_{N\;B34}(z)\right)}_{\mathrm{AbSc}}=\beta_{B34}\int_{\lambda 3}^{\lambda 4}\alpha (\lambda )\varphi_{N\;B}(\lambda )(F(\lambda )e^{-\mu_{e}(\lambda )z}+G(\lambda )e^{-k_{d}(\lambda )z})d\lambda ,$$ (19)

$${\left(\Phi_{N\;B12}(z)\right)}_{\mathrm{AbSc}}=\beta_{B12}\int_{\lambda 1}^{\lambda 2}\alpha (\lambda )\varphi_{N\;B}(\lambda )(F(\lambda )e^{-\mu_{e}(\lambda )z}+G(\lambda )e^{-k_{d}(\lambda )z})d\lambda ,$$ (20)

where $\beta_{A34}=\frac{\Phi_{N0\;A34}}{\int_{\lambda 3}^{\lambda 4}\alpha (\lambda )\varphi_{N\;A}(\lambda )(F(\lambda )+G(\lambda ))d\lambda }$, $\beta_{B34}=\frac{\Phi_{N0\;B34}}{\int_{\lambda 3}^{\lambda 4}\alpha (\lambda )\varphi_{N\;B}(\lambda )(F(\lambda )+G(\lambda ))d\lambda }$ and $\beta_{B12}=\frac{\Phi_{N0\;B12}}{\int_{\lambda 1}^{\lambda 2}\alpha (\lambda )\varphi_{N\;B}(\lambda )(F(\lambda )+G(\lambda ))d\lambda }$ are constant.

The extinction coefficient $\mu_{e}(\lambda )$ can be expressed as the product of $\mu_{t}^{\prime }(\lambda )$ by the coefficient of proportionality, $a_{e}$. Reformulating Eqs. (15)–(16) and Eqs. (18)–(20) gives Eqs. (27)–(31) in Appendix B.

Equation (17) converges toward ${\left(\frac{N_{\lambda 3\;\lambda 4}}{N_{\lambda 1\;\lambda 2}}\right)}_{Ab}$ [Eq. (10)] when scattering decreases toward zero.

At large depth, when $z\gg \frac{1}{\mu_{e}(\lambda )}$, Eq. (17) converges toward $\frac{\frac{[A]\sigma_{N}}{\left[B\right]}\beta_{A34}\int_{\lambda 3}^{\lambda 4}\alpha (\lambda )\varphi_{NA}(\lambda )G(\lambda )e^{-k_{d}(\lambda )z}d\lambda }{\beta_{B12}\int_{\lambda 1}^{\lambda 2}\alpha (\lambda )\varphi_{NB}(\lambda )G(\lambda )e^{-k_{d}(\lambda )z}d\lambda }+\frac{\beta_{B34}\int_{\lambda 3}^{\lambda 4}\alpha (\lambda )\varphi_{NB}(\lambda )G(\lambda )e^{-k_{d}(\lambda )z}d\lambda }{\beta_{B12}\int_{\lambda 1}^{\lambda 2}\alpha (\lambda )\varphi_{NB}(\lambda )G(\lambda )e^{-k_{d}(\lambda )z}d\lambda }$, which corresponds to depth-dependent exponential attenuation governed by the wavelength-dependent $k_{d}(\lambda )$ attenuation coefficient.

According to our AbSc model, after propagation through the medium, the ratio of collected photons emitted in the two spectral bands is therefore determined by the system optical transmission, the fluorophores concentrations, their photochemical properties, and the wavelength-dependence of absorption and scattering in the medium.

The AbSc model can be used directly when the optical parameters and cytoarchitecture are homogeneous within the medium. Interestingly, it can also be used to analyze multilayer structures, provided that each layer is homogeneous. In these conditions, the AbSc model must be calculated for each layer, starting from the outermost layer. The attenuation of the fluorescence ratio is then given by the product of the attenuation of the AbSc model in each layer. For example, in a two-layer structure (L1 and L2), where the interface is located at a depth of $z_{0}$, the fluorescence ratio at a depth of z is given by the following product:

$$\frac{{\left(\frac{N_{\lambda 3\;\lambda 4}}{N_{\lambda 1\;\lambda 2}}\right)}_{AbSc}(z)}{{\left(\frac{N_{\lambda 3\;\lambda 4}}{N_{\lambda 1\;\lambda 2}}\right)}_{AbSc}(0)}={\left(\frac{{\left(\frac{N_{\lambda 3\;\lambda 4}}{N_{\lambda 1\;\lambda 2}}\right)}_{AbSc}\left(z_{0}\right)}{{\left(\frac{N_{\lambda 3\;\lambda 4}}{N_{\lambda 1\;\lambda 2}}\right)}_{AbSc}\left(0\right)}\right)}_{L1}*{\left(\frac{{\left(\frac{N_{\lambda 3\;\lambda 4}}{N_{\lambda 1\;\lambda 2}}\right)}_{AbSc}\left(z-z_{0}\right)}{{\left(\frac{N_{\lambda 3\;\lambda 4}}{N_{\lambda 1\;\lambda 2}}\right)}_{AbSc}\left(z_{0}\right)}\right)}_{L2}.$$ (21)

### 3.2 Absorption and Scattering Coefficients in the Cerebral Cortex and Its Vessels

As our study relies on intravascular imaging to investigate dual-color ratiometric measurements in the cerebral cortex, it was necessary to characterize the wavelength dependence of the optical properties of the cerebral cortex and of blood vessels (Fig. 2).

**Fig. 3:**
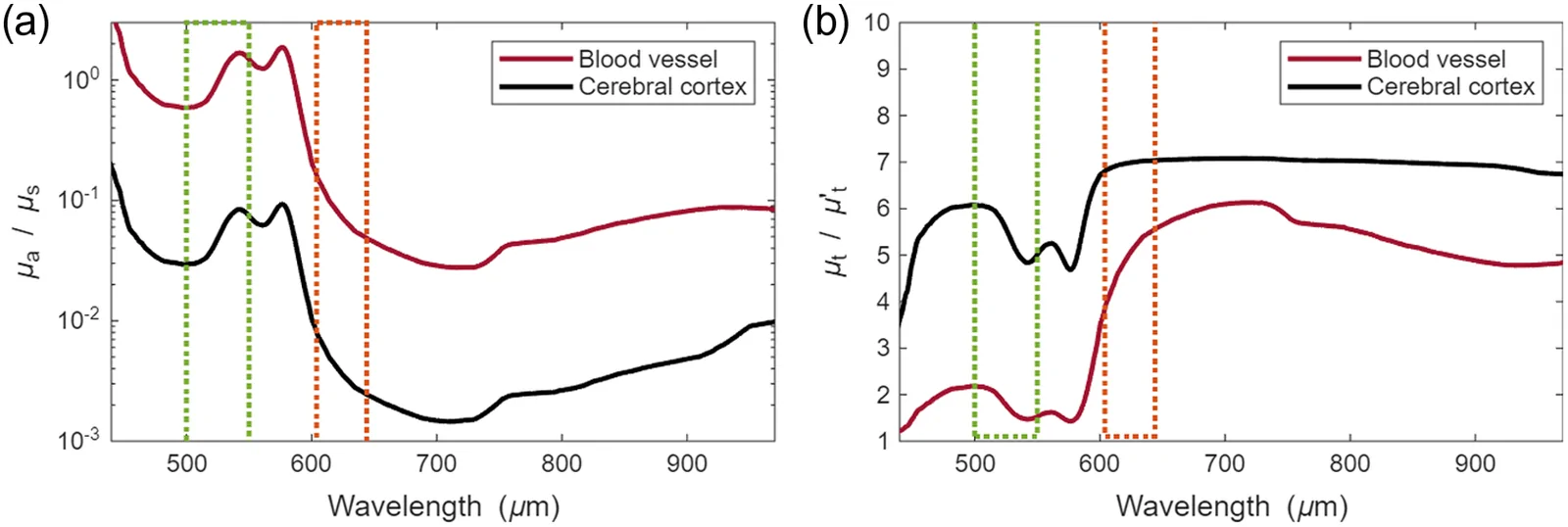
Wavelength dependence of attenuation ratios. (a) Ratio of the absorption coefficient by the scattering coefficient. (b) Ratio of the total attenuation coefficient by the reduced total coefficient for each medium. The spectral bands used in the simulations are highlighted using the corresponding colors.

The absorption and scattering coefficients, as well as the scattering anisotropy factor, have been extensively measured in human postoperative or postmortem cortical tissues^49^^,^^50^^–^^53^ and in pressurized pieces of rodent brain tissue.^54^^,^^55^ These studies consistently report a decrease of both the absorption and scattering coefficients with increasing wavelength, accompanied by an increase in the anisotropy factor. However, substantial differences in optical parameters exist between human and mouse brains, as well as across brain regions, depending on cytoarchitecture, particularly myelinization, cell size, and vascular density.^56^^–^^59^

*In vivo* measurements of absorption and scattering coefficients in the rodent cortex have been reported only at a limited number of wavelengths.^17^^,^^57^^,^^60^^,^^61^ Additional estimates at a few wavelengths have been obtained using multispectral imaging^62^ or reflectance spectroscopy.^63^ Consequently, the wavelength dependence of absorption and scattering coefficients in the living mouse cerebral cortex cannot be directly derived from available experimental data. We therefore computed the wavelength dependence of the absorption coefficient from its individual components and modeled the wavelength dependence of the scattering coefficient.

We first computed the blood absorption coefficient as the sum of contributions from the main absorbing compounds,^8^ namely, oxygenated or deoxygenated hemoglobin, within the 400 to 900 nm spectral range. Accordingly, the blood absorption coefficient was computed as

$$\mu_{a\;\mathrm{Blood}}(\lambda )=[\mathrm{HbT}]({\mathrm{SO}}_{2}\mu_{a\;HbO2}\left(\lambda \right)+\left(1-{\mathrm{SO}}_{2}\right)\mu_{a\;Hb}\left(\lambda \right)),$$ (22)

where [HbT] is the total hemoglobin concentration in blood, $[\mathrm{HbT}{]}_{\text{Mouse}}=2.3\;\mathrm{mM}$^64^ and ${\mathrm{SO}}_{2}$ is hemoglobin saturation in oxygen. In mouse cortical vessels, ${\mathrm{SO}}_{2}\approx 0.8$^65^ when the objective is not heated. The absorption spectra of oxy- and deoxy- hemoglobin, $\mu_{a\;\mathrm{HbO}}$ and $\mu_{a\;\mathrm{Hb}}$ were taken from.^10^ The absorption spectra shown in Fig. 2 indicate that the wavelength-dependent relative changes in blood absorption are governed by the combined contributions of oxyhemoglobin and deoxyhemoglobin.

The absorption coefficient of the mouse cerebral cortex was calculated as the sum of the contributions from blood ($\mu_{a\;\mathrm{Blood}}$), water ($\mu_{a\;\mathrm{Water}}$) and lipid ($\mu_{a\;\mathrm{Lipid}}$).^8^

$$\mu_{a\;\mathrm{Cortex}}(\lambda )=\chi_{\mathrm{Blood}\;\mathrm{CC}}\mu_{a\;\mathrm{Blood}}(\lambda )+\chi_{\mathrm{Water}\;\mathrm{CC}}\mu_{a\;\mathrm{Water}\;}(\lambda )+\chi_{\mathrm{Lipid}\;\mathrm{CC}}\mu_{a\;\mathrm{Lipid}\;}(\lambda ).$$ (23)

Here $\chi_{\mathrm{Blood}\;\mathrm{CC}}$, $\chi_{\mathrm{Water}\;\mathrm{CC}}$, and $\chi_{\mathrm{Lipid}\;\mathrm{CC}}$ denote the volume fractions of blood vessels, water, and lipids within the cortical volume traversed by emitted photons propagating from the focal point to the objective (Fig. 1). The average fractional blood volume in the cortex has been reported to range from 1% to 4%,^66^^–^^72^ with a median at ∼2.5%. However, cortical layer 1, exhibits a fractional volume of vasculature approximatiely twice that of deeper layers^67^ and plays a critical role, as all the collected photons must traverse this layer. Accordingly, to estimate the wavelength dependence of $\mu_{a\;\mathrm{Cortex}}$ and perform initial numerical simulations, we used $\chi_{\mathrm{Blood}\;\mathrm{CC}}=5\%$, corresponding to twice the median of the reported cortical values. Nevertheless, at the spatial scale of 2-photon microscopy, blood vessel density varies substantially depending on the presence or absence of large surface vessels. Therefore, in subsequent experimental analysis, $\chi_{\mathrm{Blood}\;\mathrm{CC}}$ was treated as a variable parameter and optimized for each cortex investigated. We used $\chi_{\mathrm{Water}\;\mathrm{CC}}=0.8$.^73^^,^^74^ The wavelength dependance of $\mu_{a\;\mathrm{Water}}$ was compiled form Refs. 75–77. The lipid volume fraction was estimated to 0.12,^74^ and the wavelength dependance of $\mu_{a\;\mathrm{Lipid}}$ was obtained from.^78^

In the cerebral cortex (Fig. 2), wavelength-dependent relative changes in absorption due to lipids and water are negligible compared with those arising from hemoglobin at wavelengths below 700 nm.

The wavelength dependence of $\mu_{s}$ was modelled using Mie scattering theory:^8^

$$\mu_{s}(\lambda )=a_{\text{cortex}}{\left(\frac{\lambda }{500}\right)}^{-b_{\text{cortex}}}.$$ (24)

The parameters were optimized using published experimental data (Table 1), yielding $a_{\text{cortex}}=0.019\pm 0.002$ and $b_{\text{cortex}}=1.6\pm 0.4$ (95% confidence interval) with a coefficient of determination $R^{2}=0.95$ (Fig. 2).

**Table 1:** Summary of reported values of $\mu_{s}(\lambda )$ in the cerebral cortex from^17^^,^^57^^,^^60^^,^^61^ at specific wavelengths, obtained from acute brain slices and *in vivo* studies.

Reference	Type	λ (nm)	$\mu_{t=}\mu_{a}+\mu_{s}$ $\left(\mu m^{-1}\right)$	$\mu_{a}$ $\left(\mu m^{-1}\right)$	$\mu_{s}$ $\left(\mu m^{-1}\right)$
60	Acute brain slices	473	$2.11\times 10^{-2}$	0	$2.11\times 10^{-2}$
57	Acute brain slices	532	$1.60\times 10^{-2}$	0	$1.60\times 10^{-2}$
17	Acute brain slices	725	$1.29\times 10^{-2}$	0	$1.29\times 10^{-2}$
17	Acute brain slices	800	$9.64\times 10^{-3}$	0	$9.64\times 10^{-3}$
17	Acute brain slices	850	$7.28\times 10^{-3}$	0	$7.28\times 10^{-3}$
17	Acute brain slices	900	$6.68\times 10^{-3}$	0	$6.68\times 10^{-3}$
61	In vivo	920	$6.45\times 10^{-3}$	$4\times 10^{-5}$	$6.41\times 10^{-3}$
17	Acute brain slices	950	$6.08\times 10^{-3}$	$6\times 10^{-5}$	$6.02\times 10^{-3}$

The scattering anisotropy factor $g_{s}$ has been reported to be 0.86 at 473 nm^60^ and approximately 0.9 at 405, 532 and 635 nm^57^ in living rodent cortical tissue. These observations indicate that scattering anisotropy is relatively weakly dependent on wavelength in the visible range, consistent with postmortem grey matter.^55^ Consequently, we assumed $g_{s}$ to be wavelength-independent in living rodent cortical tissue and equal to 0.86.

Using the wavelength dependence of $\mu_{a}$ and $\mu_{s}$, together with the constant anisotropy factor $g_{s}$, we derived the wavelength dependence of the reduced scattering coefficient $\mu_{s}^{\prime }$, the total attenuation coefficient $\mu_{t}$, and the reduced total attenuation coefficient $\mu_{t}^{\prime }$. These quantities were calculated for both individual blood vessels and the cerebral cortex (Fig. 2). The resulting spectra closely matched those previously estimated by^62^ and ^63^ from a limited set of wavelengths, thereby validating our approach. Overall, the absorption and scattering coefficients in the rodent cerebral cortex exhibit strong wavelength dependence in the visible range and can significantly affect the depth dependence of the fluorescence ratio.

### 3.3 Impact of Wavelength-Dependence of Absorption and Scattering Coefficients on Dual Color Ratiometric Measurements

#### 3.3.1 Mathematical simulation of the impact of wavelength-dependence of absorption and scattering on dual color ratiometric measurements in a blood vessel and in cerebral cortex tissue

We next simulated the impact of wavelength-dependent absorption and scattering on the depth-dependence of dual-color ratiometric measurements in both individual vessels and the cerebral cortex. Typical experimental conditions were considered, involving two commonly used fluorophores – one emitting in the green and the other in the red spectral range – and signal detection in corresponding green and red spectral bands (see Sec. 2.1 for details).

Within blood vessels, in the green spectral band, the absorption and scattering coefficients are of comparable magnitude [Fig. 3(a)]. As a result, absorption is expected to strongly affect light propagation. In the red spectral band, the absorption coefficient is approximately one order of magnitude lower than the scattering coefficient. Therefore, although absorption remains non-negligible, more scattering is expected to occur [Fig. 3(a)].

**Fig. 4:**
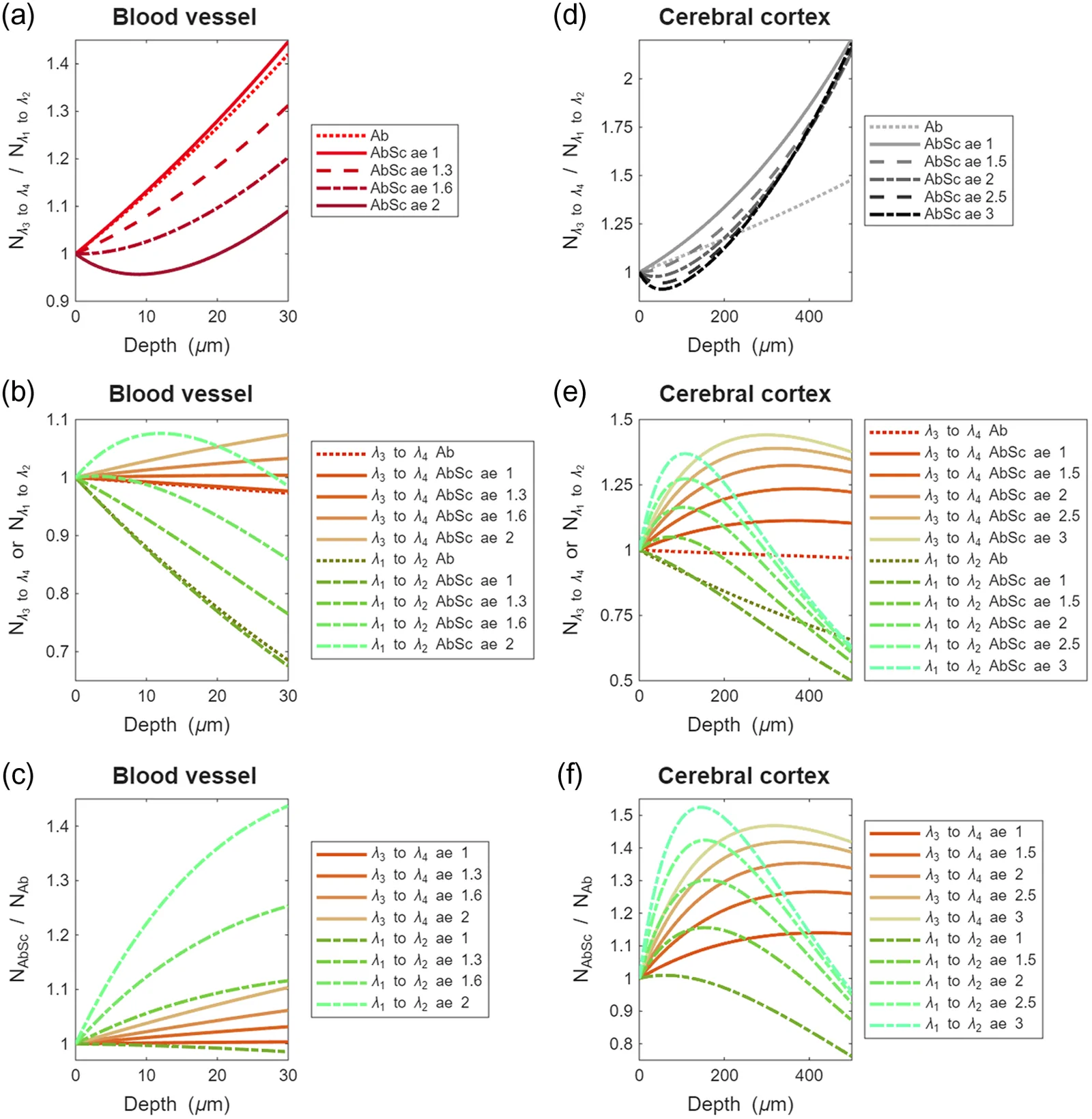
Modeling the impact of wavelength-dependent absorption and scattering on ratiometric imaging. (a) Simulation of the absorption-only [Ab, Eq. (10)] and the absorption and scattering [AbSc, Eq. (17)] models over a range of $a_{e}$ coefficients. (b) Simulation of the depth dependence of the photon number (normalized to the surface value) for each wavelength range for each model. (c) Simulation of depth-dependent ratio between the absorption and scattering model and the absorption-only model. Panels (d)–(f) show the same as panels (a)–(c) applied to ratiometric imaging to cerebral cortex with $\chi_{\mathrm{Blood}\;\mathrm{CC}}=5\%$.

In the cerebral cortex, the scattering coefficients exceed the absorption coefficients by one to two orders of magnitude across both spectral ranges [Fig. 3(a)]. Consequently, and contrary to common assumptions in the literature,^19^^,^^79^ both absorption and scattering are expected to significantly influence dual color ratiometric measurements in cortical tissue.

We performed numerical simulations with the Ab model [Eq. (10)] and our AbSc model [Eq. (17)] for both the blood vessel and the cerebral cortex. For the AbSc model, the values of *ae* are unknown. To estimate their order of magnitude, we computed the wavelength dependence of the ratio $\mu_{t}/\mu_{t}^{\prime }$ in blood vessels and in the cerebral cortex [Fig. 3(b)]. In blood vessels, this ratio remains below 2 in the green spectral band and below 6 in the red spectral band, suggesting that $a_{e}$ is expected to be between 1 and 2. In the cerebral cortex, the ratio $\mu_{t}/\mu_{t}^{\prime }$ varies between 5 and 7, so $a_{e}$ is expected to be between 1 and 5.

We investigated depths of up to 30  μm within individual blood vessels, corresponding to the diameter of a cortical arteriole. At these depths, absorption alone produces a pronounced increase in the red-to-green ratio of collected photons with depth [Fig. 4(a)]. This behavior is expected, as green photons are absorbed more strongly than red photons. At a depth of 30  μm, the red-to-green ratio reached 140% of its surface value and therefore cannot be neglected. The inclusion of scattering slightly enhances this depth-dependent increase when $a_{e}=1$, but progressively attenuates it for larger values of $a_{e}$ [Fig. 4(a)]. This attenuation becomes more pronounced as $a_{e}$ increases. To elucidate this effect, we examined the depth dependence of the number of collected photons in each wavelength band separately. As expected, for the absorption-only model, the number of photons in the green channel decreases steeply with depth [Fig. 4(b)]. When scattering is included, this decrease is accentuated for $a_{e}=1$, but reduced for $a_{e}>1$. Indeed, scattering can increase the number of collected photons at shallow depth, as previously reported under different conditions.^9^ As $a_{e}$ increases, scattering becomes more isotropic, thereby enhancing this effect. In contrast, absorption has little effect on the depth-dependence of the number of red photons Fig. 4(b). Scattering induces a slight increase in the number of collected red photons with depth, up to 30  μm. As for green photons, the magnitude of this increase is correlated with the value of $a_{e}$. However, relative to absorption alone, this scattering-induced increase [Fig. 4(c)] is much larger for the green wavelength band than for the red wavelength band [Fig. 4(c)]. Consequently, scattering attenuates the depth-dependent absorption-induced increase in the red-to-green fluorescence ratio, with the extent of this attenuation depending on $a_{e}$ [Fig. 4(a)].

**Fig. 5:**
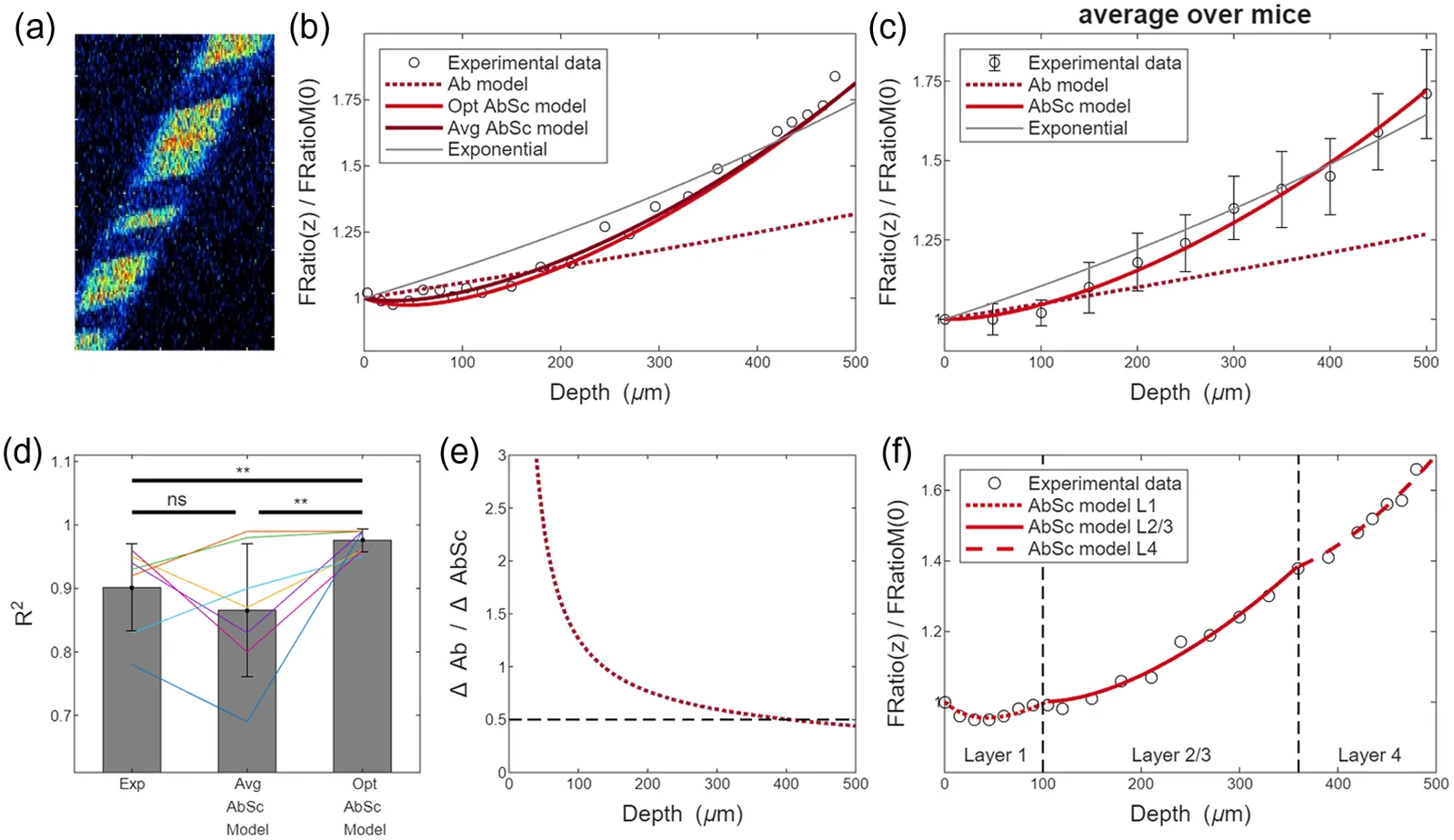
Experimental assessment of the impact of wavelength-dependent absorption and scattering on dual-color ratiometric measurements in the cortex. (a) Two-photon image of a representative capillary in which measurements were performed. Red blood cells appear as dark shadows within fluorescent plasma. (b) FRatio(z) was plotted as a function of depth and fitted with the AbSc model using experiment-specific optimized $a_{e}$ and $\chi_{\mathrm{Blood}\;\mathrm{CC}}$ values (Opt AbSc model), the AbSc model using population-averaged $a_{e}$ and $\chi_{\mathrm{Blood}\;\mathrm{CC}}$ values across mice (Avg AbSc model), the absorption-only model (Ab model), and a simple exponential. (c) Same analysis as (b) using data averaged across mice (n=7 mice). (d) Statistics of the coefficient of determination ($R^{2}$). Individual mice (colored lines) and average (grey bars) (n=5 mice). **p<0.001, nonparametric Wilcoxon signed-rank paired test. (e) Ratio between the increase in FRatio(z)/FRatio M(0) attributable to absorption alone and the increase attributable to combined absorption and scattering. (f) FRatio(z) was plotted as a function of depth and fitted with the AbSc model using experiment-specific optimized $a_{e}$ and $\chi_{\mathrm{Blood}\;\mathrm{CC}}$ values (Opt AbSc model) for each cortical layer (average of n=2 mice). Data are presented as mean ± stdev.

To summarize, the depth dependence of the red-to-green fluorescence ratio within a blood vessel can introduce bias when the depth dependence of this ratio is investigated in the cerebral cortex. To minimize interference from hemoglobin in red blood cells, experiments examining the depth dependence of the vascular red-to-green fluorescence ratio in the cerebral cortex should focus on the smallest vessels, such as capillaries.

For the cerebral cortex, we examined depths up to 500  μm, which is typical for 2-photon imaging. Absorption alone resulted in a pronounced increase in the red-to-green ratio of collected photons with depth [Fig. 4(d)], reaching ∼150% at 500  μm. When scattering was included, this depth-dependent increase was further enhanced for $a_{e}=1$ [Fig. 4(d)]. Larger values of $a_{e}$, corresponding to more isotropic scattering, induced complex effects, including a transient decrease in the red-to-green ratio near the surface followed by an increase at greater depths. The magnitude of these effects scaled with the value of $a_{e}$. However, regardless of $a_{e}$, the red-to-green ratio of collected photons reached ∼220% of its surface value at a depth of 500  μm. To better understand these observations, we again analyzed the depth dependence of the number of collected photons in each wavelength band. As with individual blood vessels, the number of photons in the green wavelength band decreases steeply with depth in the absorption-only model [Fig. 4(e)]. The inclusion of scattering mitigates absorption losses by increasing the number of detected photons. This effect is strongest near the surface, and its amplitude increases with the value of $a_{e}$. In the absorption-only case, the number of red photons decreases only slightly with depth [Fig. 4(e)]. When scattering is included, the number of red photons increases with depth, with an amplitude that correlates with $a_{e}$. Compared with absorption alone, scattering induces a larger increase in green photons than in red photons up to depths of 250  μm. Consequently, the addition of scattering reduces the depth-dependent increase in the red-to-green fluorescence ratio [Fig. 4(f)]. At greater depths, however, the scattering-induced increase in red photons exceeds that of green photons. As a result, the depth-dependent increase in the red-to-green photon ratio is enhanced when scattering is taken into account.

#### 3.3.2 Experimental testing of the depth-dependence of dual color ratiometric measurements in the cerebral cortex

Next, we tested our mathematical models experimentally by imaging the blood vessels of mice whose blood plasma had been labeled with fluorescent molecules (see Sec. 2 for details). The red-to-green fluorescence ratio was measured under the same imaging conditions as those described in Sec. 3.3.1.

We first quantified the dependence of the red-to-green fluorescence ratio as a function of cortical depth. To sample this ratio independently of any depth-dependent effects within individual vessels, we imaged the smallest blood vessels (i.e., capillaries), in which red blood cells (RBCs) occupy the entire capillary diameter, as explained in Sec. 3.3.1. To minimize interference from hemoglobin absorption in RBCs in the imaged capillary, we selected horizontally oriented capillary segments [Fig. 5(a)]. The red-to-green fluorescence ratio was measured as a function of both depth and time, and TDCCF correction was performed (Sec. 2.3 and Fig. 11 in Appendix A for details). In each experiment, the red-to-green fluorescence ratio increases clearly with depth and was well fitted by our AbSc model [Fig. 5(b)], with a coefficient of determination $R^{2}=0.98\pm 0.02$ (n=7 mice), yielding $a_{e}=1.8\pm 0.6$ (mean ± stdev, n=7 mice) and $\chi_{\mathrm{Blood}\;\mathrm{CC}}=3.5\pm 0.8\%$ (mean ± stdev, n=7 mice). The average red-to-green fluorescence ratio across all experiments was also well described by the AbSc model [Fig. 5(c)] with $R^{2}=0.99$, yielding $a_{e}=1.5$ and $\chi_{\mathrm{Blood}\;\mathrm{CC}}=3\%$. These results demonstrate that the AbSc model provides an accurate description of experimental data obtained in the cerebral cortex.

**Fig. 6:**
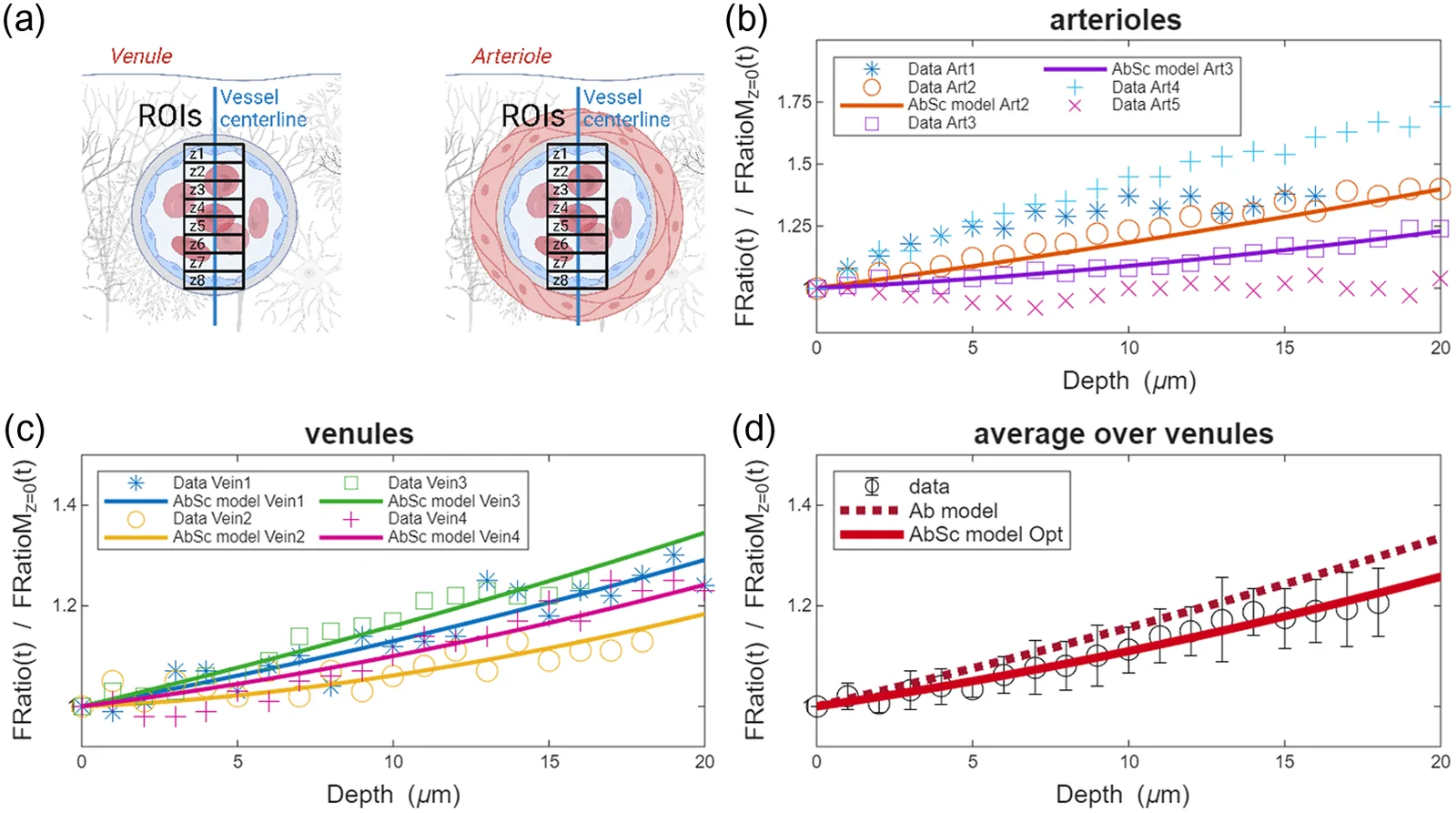
Experimental evaluation of the impact of wavelength-dependent absorption and scattering on dual-color ratiometric measurements within blood vessels. (a) Schematic illustrating the regions of interest (ROIs) used to analyze $\text{FRatio}/{\text{FRatio}}_{z=0}$ in venules and arterioles. (b) $\text{FRatio}/{\text{FRatio}}_{z=0}$ as a function of depth from five arterioles, with fits using the optimized AbSc model when applicable. (c) Same as (b) for venules. (d) Mean $\text{FRatio}/{\text{FRatio}}_{z=0}$ across venules plotted versus depth (mean ± stdev, n=4 venules), together with fits using the optimized AbSc model and the absorption-only (Ab) model.

**Fig. 7:**
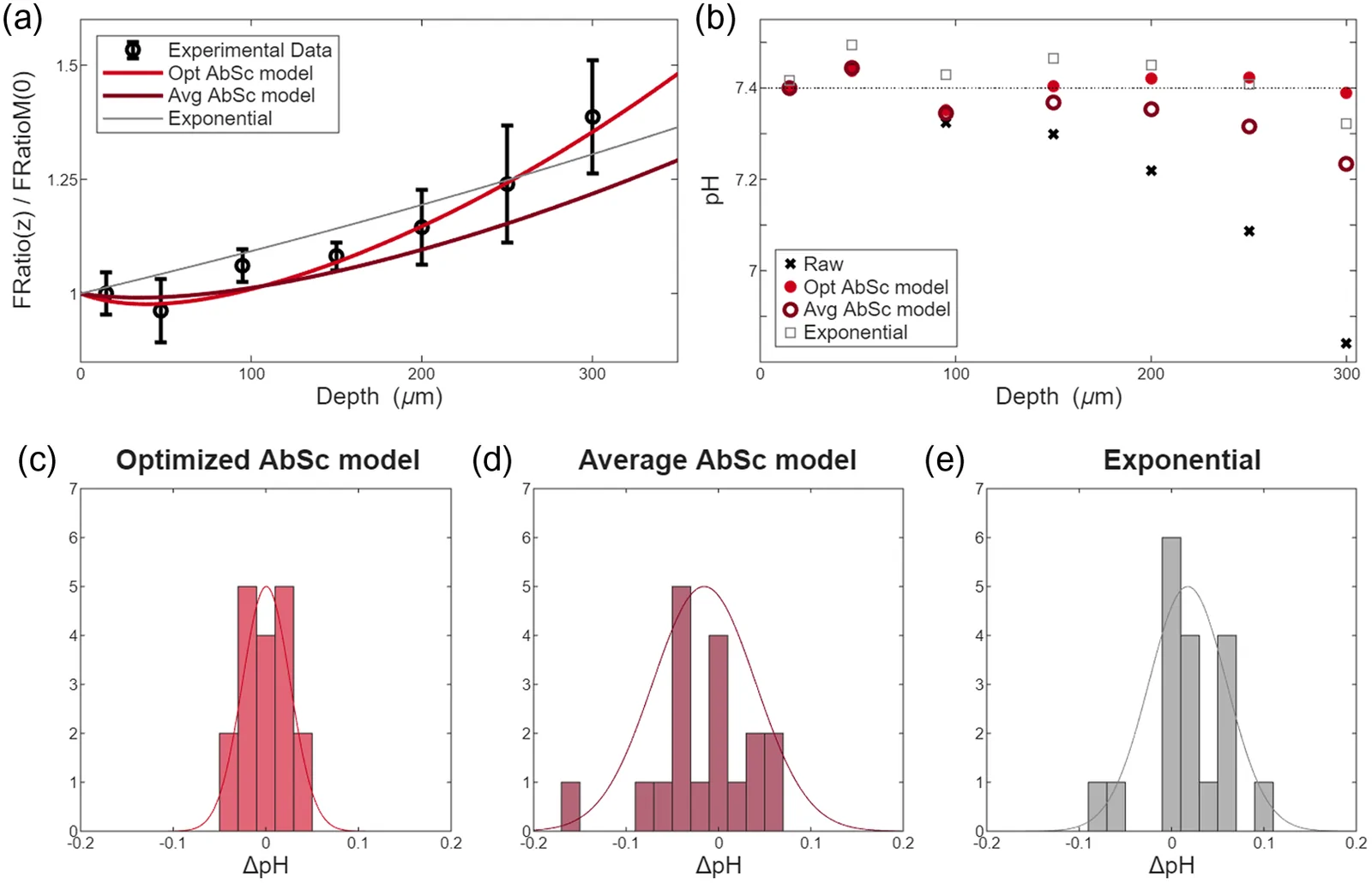
Experimental evaluation of the impact of wavelength-dependent absorption and scattering on intravascular pH measurements. (a) The depth-dependent average FRatio(z)/FRatio M(0) obtained from a single experiment (mean ± stdev, n=3 time points) was fitted with the optimized AbSc model, the average AbSc model, and an exponential model. (b) pH values were computed from data from panel (a) using model-specific corrections. Panels (c)–(e) show distribution of residual pH error after correction with each of the models together with Gaussian fits.

Finally, for each experiment, we compared model fits obtained using mouse-specific optimized values of $a_{e}$ and $\chi_{\mathrm{Blood}\;\mathrm{CC}}$ with fits obtained using the average values of these parameters across all mice. Using the average parameters still resulted in good fits [$R^{2}=0.87\pm 0.1$ (n=7), Figs. 5(b) and 5(d)], indicating that an average AbSc model can be used to estimate the depth dependence of the red-to-green fluorescence ratio in the cortex. However, this approach was less accurate than using mouse-specific optimized parameters (optimized AbSc model), which yielded significantly higher coefficients of determination (p<0.001, Wilcoxon signed-rank paired test) for average parameters [Fig. 5(d)].

We next compared the AbSc model with a simple exponential fit. The exponential model provided a good fit [$R^{2}=0.9\pm 0.06$ (n=7), Fig. 5(d)]. Although its performance was comparable to that of the average AbSc model, it remained less accurate than the optimized AbSc model [Fig. 5(d)]. Moreover, both the average and optimized AbSc models consistently yielded better fits at intermediate depths [Figs. 5(b) and 5(c)]. Overall, the optimized AbSc model outperformed the simple exponential fit. Furthermore, our average AbSc model performed as well as the exponential fit while not requiring any depth-dependent calibration.

We further examined the contribution of absorption alone to the depth-dependent increase in the red-to-green fluorescence ratio [Fig. 5(e)]. As anticipated from Sec. 3.3.1, at shallow depths, the increase in the red-to-green fluorescence attributable solely to absorption greatly exceeded that arising from the combined effects of absorption and scattering. At larger depths, however, absorption alone still accounted for approximately half of the total increase in the red-to-green fluorescence ratio. These results demonstrate that absorption plays a fundamental role in the optical modeling of emitted light in the cerebral cortex and cannot be neglected.

We then tested a multi-layer analysis, exploring cortical depths up to 500 micrometers, comprising three layers according to the cytoarchitecture and the Allen brain atlas: layer 1 going from the surface to ∼100 micrometers in depth, layer 2/3 going from ∼100 micrometers in depth to ∼360 micrometers, and layer 4 from ∼360 to ∼500 micrometers. In the two explored mice, the relationship between depth and red-to-green fluorescence ratio in each layer, could be fitted with the AbSc model [Fig. 5(f)], giving: in layer 1 $a_{e}=2.5\pm 0.6$ and $\chi_{\mathrm{Blood}\;\mathrm{CC}}=6\pm 1\%$ (mean ± stdev, $R^{2}=0.6\pm 0.2$, n=2 mice), in layer 2/3 $a_{e}=1.3\pm 0.2$ and $\chi_{\mathrm{Blood}\;\mathrm{CC}}=5.0\pm 0.7\%$ (mean ± stdev, $R^{2}=0.97\pm 0.00$, n=2 mice), and in layer 4 $a_{e}=1.5\pm 0.1$ and $\chi_{\mathrm{Blood}\;\mathrm{CC}}=7\pm 1\%$ (mean ± stdev, $R^{2}=0.99\pm 0.01$ n=2 mice). Parameter $a_{e}$ was larger in layer 1 than in layer 2/3 and layer 4, suggesting that scattering is more isotropic at the brain surface than at the interface between underlying cortical layers. Overall, these results demonstrate that our model can be successfully applied using a multi layer framework.

#### 3.3.3 Experimental testing of dual-color ratiometric measurements in the cerebral cortex vessels and below

Finally, we experimentally evaluated our AbSc model on intravascular imaging. We examined a set of arterioles and venules on the surface of the mouse cortex, with diameters ranging from 16 to 51  μm. As scattering is limited within blood vessels [see Sec. 3.3.1 and Fig. 4(a)], the propagation of emitted photons is expected to be predominantly in the forward direction. Therefore, the collected fluorescence can be approximated by the cone of excitation light within the blood vessels (see Fig. 12 in Appendix E). Under this assumption, fluorescence photons emitted along the vessel centerline traverse similar path lengths within the vessel cross-section (Fig. 12 in Appendix E). In contrast, fluorescence photons emitted off-axis experience the much wider range of path lengths before collection (Fig. 12 in Appendix E). Consequently, the depth-dependance of the red-to-green fluorescence ratio is expected to be sensitive to the choice of the ROI. To test this hypothesis, we compared ROIs centered on the vessel centerline with ROIs positioned away from the centerline (Fig. 12 in Appendix E)in a set of venules. For each vessel, the red-to-green fluorescence ratio exhibited a substantially stronger depth dependent increase in centerline ROIs than on off-center ROIs (Fig. 12 in Appendix E), demonstrating that ROI selection has a major impact on the measured signal and resulting analysis.

**Fig. 8:**
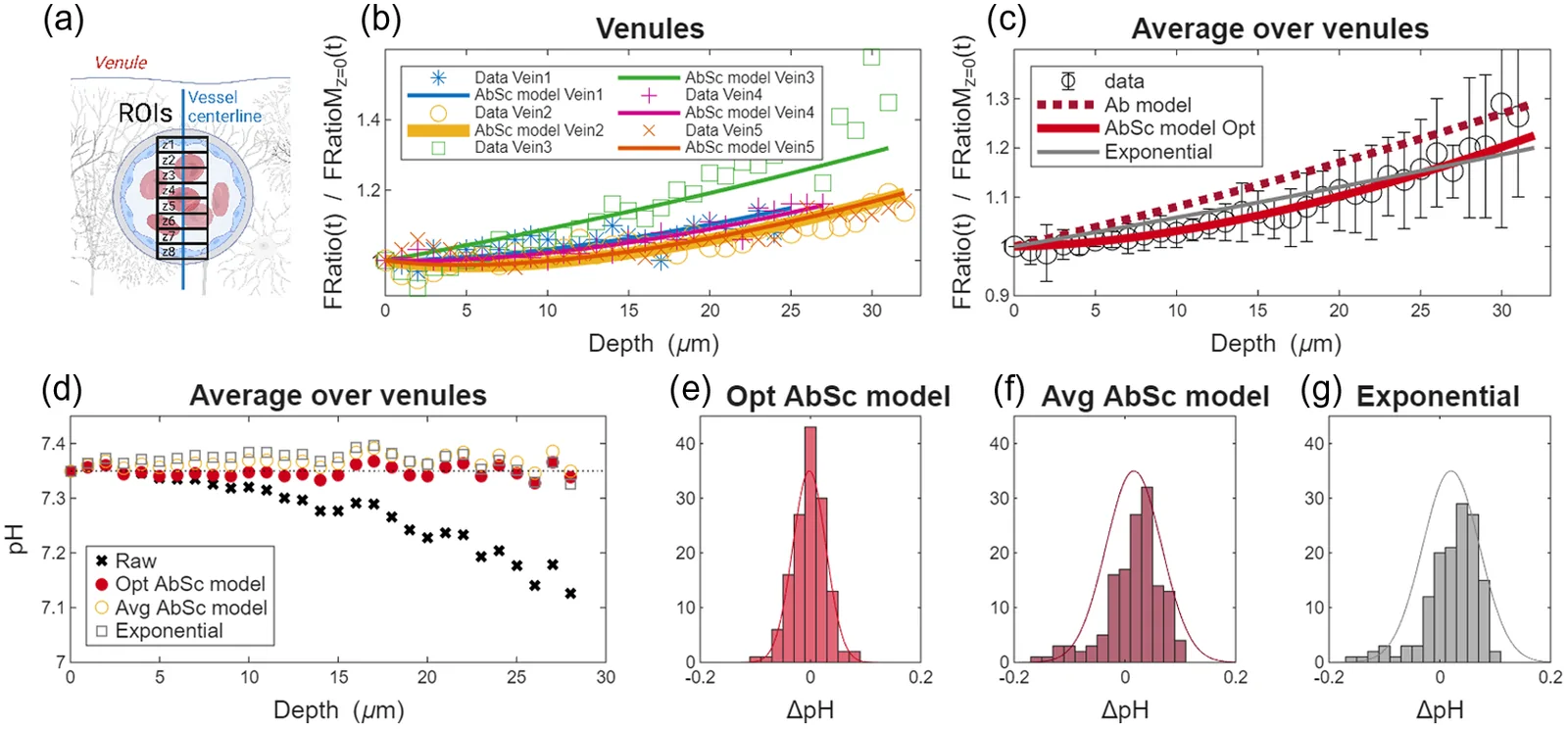
Experimental evaluation of the impact of wavelength-dependent absorption and scattering on intravascular pH measurements. (a) Schematic illustrating the regions of interest (ROIs) used to analyze FRatio(z)/FRatio(0) in venules. (b) The depth-dependent FRatio(z)/FRatio(0) obtained from each venule was fitted with the optimized AbSc model. (c) The average depth-dependent FRatio(z)/FRatio(0) over venules (mean ± stdev, n=5 venules) was fitted with the optimized AbSc model, the Ab model and an exponential model. (d) pH values were computed from data from panel (a) using model-specific corrections for each venule and then averaged. Panels (e)–(g) show distribution of residual pH error after correction with each of the models together with Gaussian fits.

**Fig. 9:**
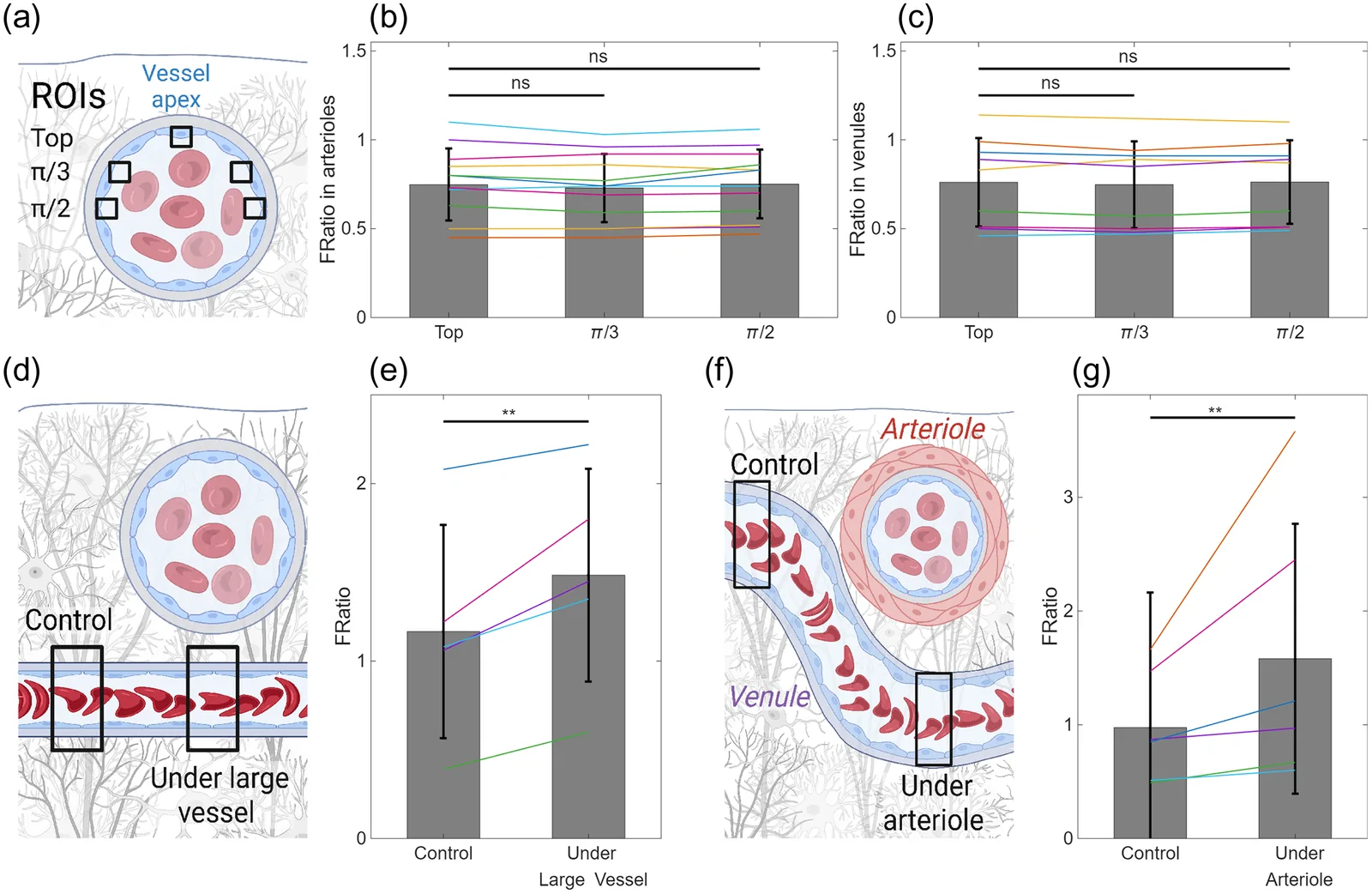
Effect of large vessels on ratiometric measurements taken on their sides and beneath them. Panels (a)–(c) show that red-to-green fluorescence ratio (FRatio) was compared at the top of large vessels and at various positions on their sides in arterioles (b) and in venules (c), n=12 arterioles and n=9 venules; nonparametric Wilcoxon signed-rank paired test, ns = non-significant. Panels (d)–(e) that the FRatio was compared in capillaries beneath large vessels and in control regions (mean ± stdev, n=5 capillaries). Panels (f)-(g) show that the FRatio was compared in venules going beneath arterioles and in control regions (mean ± stdev, n=6 venules, nonparametric Wilcoxon signed-rank paired test, (**) p<0.001).

**Fig. 10:**
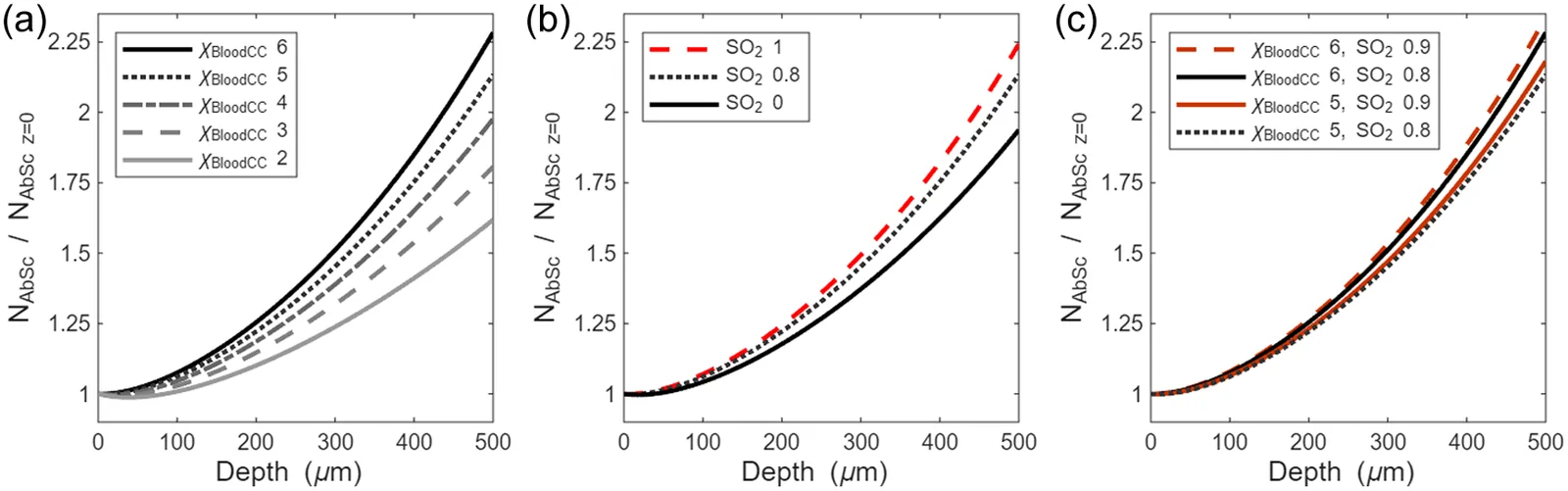
Evaluation of the impact of blood vessel density ($\chi_{\mathrm{Blood}\;\mathrm{CC}}$) and hemoglobin saturation (${\mathrm{SO}}_{2}$) on the depth dependence of the absorption and scattering model [AbSc, Eq. (17)]. (a) AbSc model for a range of $\chi_{\mathrm{Blood}\;\mathrm{CC}}$ with constant ${\mathrm{SO}}_{2}=0.8$. (b) AbSc model for a range of ${\mathrm{SO}}_{2}$ with constant $\chi_{\mathrm{Blood}\;\mathrm{CC}}=0.5$. (c) Effect of functional hyperemia on depth dependence with the AbSc model.

We applied our model to ROIs centered on the vessel centerline, where photon path lengths are most uniform, and the influence of vessel curvature is minimized [Fig. 6(a)]. In 8 out of 9 tested vessels, the red-to-green fluorescence ratio increased with depth [Figs. 6(b) and 6(c)]. A very large variability in the amplitude of this increase was observed in arterioles [Fig. 6(b)], whereas the depth-dependence was highly consistent across venules [Fig. 6(c)].

The optimized AbSc model was evaluated for each vessel individually. Only two arterioles could be successfully fitted with the optimized AbSc model ($R^{2}=0.91\pm 0.07$, n=2 arterioles, and $a_{e}=1.2\pm 0.2$). The remaining arterioles exhibited poor fits ($R^{2}<0.2$), indicating that the AbSc model is not well suited to describe arterioles. This discrepancy may arise from the presence of a highly scattering vessel wall (smooth muscle cells and lamina) surrounding arterioles, which is not accounted for in the model. In contrast, all venules were very well described by the optimized AbSc model [average $R^{2}=0.83\pm 0.12$, n=4 venules, $a_{e}=1.2\pm 0.2$, Fig. 6(c)]. The mean depth-dependence observed in venules [Fig. 6(d)] was also very well fitted by the optimized AbSc model ($R^{2}=0.95$, $a_{e}=1.25$), as well as by the absorption-only model ($R^{2}=0.91$). Although the absorption model performed slightly worse than the optimized AbSc model, the two fits were very similar, indicating that absorption accounts for most of the depth-dependent increase of the red/green fluorescence ratio in venules.

To conclude, the optimized AbSc model accurately predicts the depth dependence of the red-to-green fluorescence ratio in venules but is not well suited for arterioles.

#### 3.3.4 Application to a ratiometric biosensor in the cerebral cortex

First, we considered a biomarker M and a sensor S. Neither S nor M is fluorescent; however, their reaction product, MS, is fluorescent. As a representative example, we focused on the case where biomarker concentration is the parameter of interest. The biomarker concentration can be inferred from the fluorescence signal of the bound sensor. Using Eq. (17) and

$$[M](z)=\frac{K_{A}}{\frac{c_{S}}{\left[MS](z\right)}-1},$$ (25)

where $K_{A}$ is the equilibrium constant of the sensor-biomarker interaction, defined as $K_{A}=\frac{\left[M][S\right]}{\left[MS\right]}$, and $c_{s}$ is the total concentration of the sensor: $c_{S}=[MS]+[S]$, we obtain

$$[M](z)=\frac{K_{A}}{\frac{c_{S}\sigma_{N}{\left(\Phi_{NMS34}(z)\right)}_{AbSc}}{\left[B]({\left(\frac{N_{\lambda 3\;\lambda 4}}{N_{\lambda 1\;\lambda 2}}\right)}_{AbSc}\left([MS],[B],z\right){\left(\Phi_{NB12}(z)\right)}_{AbSc}-{\left(\Phi_{NB34}(z)\right)}_{AbSc}\right)}-1}.$$ (26)

Details to get Eq. (25) can be found in Appendix C.

We then experimentally investigated the depth dependence of a blood pH sensor in cortical tissue. Under physiological conditions, capillary blood pH is estimated to lie between 7.35 and 7.4.^80^ We performed a series of experiments under the same conditions as those described in Sec. 3.3.2, using a red-emitting pH-sensitive fluorophore with a green-emitting pH-insensitive reference fluorophore (see Sec. 2.2 for details). We measured the red-to-green fluorescence ratio as a function of depth and time, applying TDCCF correction [Fig. 7(a)]. For each experiment, we used several models to fit the depth-dependence of the red-to-green fluorescence ratio: our optimized AbSc model, our average AbSc model (using population-averaged values of $a_{e}$ and $\chi_{\mathrm{Blood}\;\mathrm{CC}}$ obtained from the previous dataset (Sec. 3.3.2)), and a simple exponential. The optimized AbSc model provided the best performance ($R^{2}=0.97\pm 0$, n=2 mice). The average AbSc model also yielded good results ($R^{2}=0.81\pm 0.14$, n=2 mice), as did the exponential model ($R^{2}=0.89\pm 0.06$, n=2 mice).

Calculation of pH as a function of depth (see Appendix D for equations), assuming a surface pH of 7.4, reveals that in the absence of a depth-dependent correction, the estimated pH appears to decrease steeply with increasing depth [Fig. 7(b)]. Applying the three correction models shown in Fig. 7(a) effectively compensates for this apparent depth-dependent pH change. Among them, the optimized absorption and scattering model provided the most accurate correction. To further compare the performance of the correction methods, we analyzed the distributions of the residual errors for each correction method using data from all mice [Figs. 7(c)–7(e)]. Again, the optimized AbSc model yielded the best correction, $\Delta {\mathrm{pH}}_{\mathrm{AbScOpt}}=0\pm 0.03$ (n=18 data points, n=2 mice). The average AbSc model resulted in a mean residual error of $\Delta {\mathrm{pH}}_{\mathrm{AbScAvg}}=-0.02\pm 0.06$ (n=18 data points, n=2 mice), while the exponential correction produced a mean residual error $\Delta {\mathrm{pH}}_{\mathrm{Exp}}=0.02\pm 0.04$ (n=18 data points, n=2 mice).

In summary, the optimized AbSc model provided the most accurate pH correction, albeit at the cost of requiring depth-dependent calibration. The average AbSc model also achieved good corrections while eliminating the need for calibration. The exponential model produced a correction of a similar quality to the average AbSc model but required depth-dependent calibration.

#### 3.3.5 Application to a ratiometric biosensor within individual vessels and beneath

We then experimentally tested the depth dependence of blood pH within single venules using the same sensor as in Sec. 3.3.4. We acquired z-stacks of individual veins and measured the red-to-green fluorescence ratio in ROIs along the vessel centerline as a function of depth only, as TDCCF is negligible in these conditions [Fig. 8(a)]. For each vessel, the depth dependence of the red-to-green fluorescence ratio was well fitted with our optimized AbSc model, ($R^{2}=0.7\pm 0.1$, n=5 venules), yielding $a_{e}=1.6\pm 0.4$. The depth dependence of the average over venules of the red-to-green fluorescence ratio was fitted with our optimized AbSc model, the Ab model, and a simple exponential. As for depth dependence within the cortex, the optimized AbSc model provided the best performance ($R^{2}=0.95$, $a_{e}=1.55$). The Ab model and the exponential fits were not good ($R^{2}=-0.94$ and 0.19, respectively). In this dataset, the range of $a_{e}$ values was broader than in the dataset used in Sec. 3.3.3, which could be due either to the larger vessels in this dataset or to the sensitivity to the choice of the ROI, as reported in Sec. 3.3.3 too.

Calculation of pH as a function of depth (see Appendix D for equations), assuming a surface pH of 7.35 within venules, reveals that, as for the cortex, in the absence of a depth-dependent correction, the estimated pH appears to decrease steeply with increasing depth [Fig. 8(d)]. Applying the three correction models shown in Fig. 8(c) effectively compensates for this apparent depth-dependent pH change. Among them, the optimized absorption and scattering model provides the most accurate correction, yielding an average residual error $\Delta {\mathrm{pH}}_{\mathrm{AbScOpt}}=0\pm 0.03$ (n=141 data points) [Fig. 8(d)]. The corrections provided by the average AbSc model and the exponential model are both slightly biased with a mean residual error of $\Delta {\mathrm{pH}}_{\mathrm{AbScAvg}}=0.02\pm 0.05$ (n=141 data points) [Fig. 8(e)] and $\Delta {\mathrm{pH}}_{\text{Exp}}=0.02\pm 0.05$ (n=141 data points) [Fig. 8(f)].

To obtain an unbiased measurement of the red-to-green fluorescence ratio in large vessels, and because measurements at the vessel apex are challenging under experimental conditions such as vasodilation, we developed an alternative method that minimizes the effects of absorption and scattering by luminal contents. Assuming that these effects would be reduced on the side of the vessel, we compared the red-to-green fluorescence ratio at the top of large vessels (both arterioles and venules) with the ratio at various positions on their sides [see Figs. 9(a)–9(c)]. We found that the ratio was not significantly different at angles inferior to or equal to π/2 for both arterioles and venules (n=12 arterioles and n=9 venules; non-parametric Wilcoxon signed-rank paired test). Therefore, in both arterioles and venules, measurements of the red-to-green fluorescence ratio obtained from ROIs adjacent to the vessel wall at angular positions ≤π/2 from the vessel apex are largely unaffected by absorption and scattering from luminal contents. Consequently, these measurements provide estimates of the intravascular red-to-green fluorescence ratio with an accuracy comparable to that obtained at the vessel apex.

We then investigated the impact of large vessels on the red-to-green fluorescence ratio measurements taken below them. We compared the ratio in capillaries beneath large vessels with that in control regions at the same depth [see Figs. 9(d)–9(e)]. Our results showed that the ratio was significantly higher beneath the large vessels than in the control regions (n=5 capillaries; p<0.001, non-parametric Wilcoxon signed-rank paired test). We also compared the red-to-green fluorescence ratio in venules passing under arterioles with that in control regions at similar depths in the cortex [Figs. 9(f)–9(g)]. To measure the ratio in the venules, we used ROIs on the side of the large vessels in their upper half, as shown in Fig. 9(a). Once again, we found that the ratio was significantly higher beneath the arterioles than in the control regions (n=6 venules; p<0.001, nonparametric Wilcoxon signed-rank paired test).

#### 3.3.6 Sensitivity to physiological parameters

We last investigated how sensitive our AbSc model is to changes in physiological parameters. As mentioned on Sec. 3.2, one of the main physiological factors contributing to the depth dependence of the AbSc model is the fractional blood volume in the cortex. Unfortunately, at the scale of multiphoton microscopy, the fractional blood volume considerably depends on the vascular anatomy of the mouse and the exact imaging location. We tested a range of physiological values and found that the depth dependence of the AbSc model can increase up to a factor of two when the fractional blood volume in the brain increases by a factor of three [Fig. 10(a)].

Another factor is the level of oxygenation of hemoglobin. We tested extreme conditions involving fully oxygenated hemoglobin and fully deoxygenated hemoglobin. We found that the depth dependence of the AbSc model is mildly sensitive to hemoglobin oxygenation. The highest depth dependence occurs when blood is fully oxygenated and the lowest when blood is fully deoxygenated [Fig. 10(b)].

Lastly, changes in neuronal activity induce functional hyperemia, which alters both the local blood volume fraction and oxygenation level. At the peak of the physiological response, these changes can reach a ∼20% increase in blood volume,^81^^–^^83^ and a local oxygen partial pressure increase of 35 to 45 mmHg,^84^^,^^85^ corresponding to an increase in ${\mathrm{SO}}_{2}$ from ∼0.8 to ∼0.9.^86^ To evaluate the impact of these changes on the depth dependence of the red-to-green fluorescence ratio, we performed numerical simulations [Fig. 10(c)]. The combined increase in the blood volume and ${\mathrm{SO}}_{2}$ change resulted in an ∼10% increase in the AbSc model prediction, with most of the effect arising from the increase in blood volume. Consequently, the accuracy of an AbSc correction computed under resting-state conditions is reduced during functional hyperemia. However, the magnitude of this error remains modest, and a resting-state correction still provides a substantially more accurate estimate than applying no correction. Whether this effect needs to be accounted for thus depends on the required measurement accuracy and could be accommodated through a simple time-dependent adjustment of the depth-dependence profile.

## 4 Discussion

We have developed an analytical model that incorporates the wavelength-dependent optical properties of biological tissues and fluorophores for ratiometric dual-color emission measurements. This model assumes homogeneous absorption and scattering parameters within a single layer or a stack of planar layers. By applying this framework to the rodent cerebral cortex and individual blood vessels, we demonstrated its ability to quantitatively describe and correct the effects of absorption and scattering in the cortex and venules. In these tissues, both absorption and scattering contribute to significantly biased dual-color ratiometric measurements. Although experiment-specific optimization of the model parameters yields the highest correction accuracy, the use of average parameters in the cortex also provides an effective, calibration-free correction. We provide a practical guide on how to use our model and perform corrections in Appendix F.

Only a limited number of models address the propagation of emitted light in the cerebral cortex with multiphoton microscopy. The model proposed by Beaurepaire and Mertz.^19^ establishes a depth dependence of collected light but does not account for wavelength-dependent absorption or scattering effects. Wang et al. ^20^ incorporated emission wavelength dependence, but modeled photon migration in the cortex using an exponential decay, an approximation that is primarily valid at large depths. Our model is the first to explicitly incorporate emission wavelength while describing the fate of emitted photons from the surface to large depths and to demonstrate the resulting consequences for ratiometric measurements, thereby filling a gap in this field.

We observed that the propagation of emitted light through the cerebral cortex significantly affects ratiometric measurements and, consequently, the estimation of physiological parameters such as pH. Our investigation showed that, when imaging the mouse cerebral cortex with two-photon microscopy, the ratio of collected red-to-green fluorescence was approximately 1.5 at depths between 300 and 400  μm. This has an impact on pH measurements of 0.5 units. This constitutes a significant error at the physiological scale and highlights the need to correct errors resulting from the absorption and scattering of emitted photons. Wang et al.^20^ reported a lower red-to-green fluorescence ratio of approximately 1.4 at greater imaging depths (600 to 800  μm) using three-photon microscopy. This difference may be explained by the fact that Wang et al. did not account for changes in plasmatic fluorophore concentration during the experiments, potentially affecting the red-to-green fluorescence ratio. Nevertheless, even the spectral distortion reported in Wang et al. is sufficient to introduce substantial errors in quantitative measurements, further emphasizing the need to correct for wavelength-dependent absorption and scattering effects in tissue imaging.

The role of absorption is often neglected^19^ or underestimated^79^ in multiphoton microscopy of biological tissues. Our results demonstrate that absorption plays a fundamental role in two-color ratiometric measurements using two-photon microscopy in the cerebral cortex. Under typical experimental conditions, involving red and green emission, absorption accounts for most of the increase of the red-to-green fluorescence ratio up to depths of ∼300  μm. A similar importance of absorption in shaping the wavelength dependance of light emerging from the cerebral cortex has been previously reported in other imaging modalities, including wide-field optical mapping (reviewed in Ref. 12) or fiber photometry.^13^ These observations underscore the necessity of carefully accounting for absorption in optical imaging of biological tissues.

Finally, we demonstrated that our model accurately describes the effects of absorption and scattering in the cerebral cortex, both when treated as a single homogeneous layer and when represented as a stack of planar layers. The model also performs well in venules but fails to adequately describe arterioles. A key distinction is that arterioles are surrounded by a thick vessel wall comprising a thick lamina and a smooth muscle cell layer, whose cells have a higher refractive index than brain tissue,^87^^,^^88^ and scatter light approximately twice as strongly as gray matter.^8^ Accurately modeling arterioles would therefore at least require the inclusion of this additional tubular scattering layer, which is not accounted for in our current framework that assumes a homogeneous distribution of scatterers. Consequently, the AbSc model is best suited to relatively homogeneous structures, such as venules, or to tissues that can be represented as a succession of planar homogeneous layers, such as the cerebral cortex. However, biological tissues are inherently heterogeneous, and local variations in cytoarchitecture, physiological properties, or the morphology and orientation of labeled structures may reduce the accuracy of the corrections. Some of these variations, as time-dependent variations, can be accounted for to improve the quality of corrections. Despite these limitations, the present findings suggest that, with appropriate parameterization, the model could be extended to a wide range of other brain regions.

## 5 Conclusion

We have developed an analytical model for dual-color ratiometric emission measurements in multiphoton microscopy that explicitly incorporates the spectral optical properties of biological tissues and fluorophores. This model enables prediction and correction of experimental measurements in the cerebral cortex and venules. Because it relies on general optical parameters, this framework is readily extensible to other biological media, including different brain regions, other organs, or other organisms.

## 6 Appendix A: TDCCF Correction

**Fig. 11:**
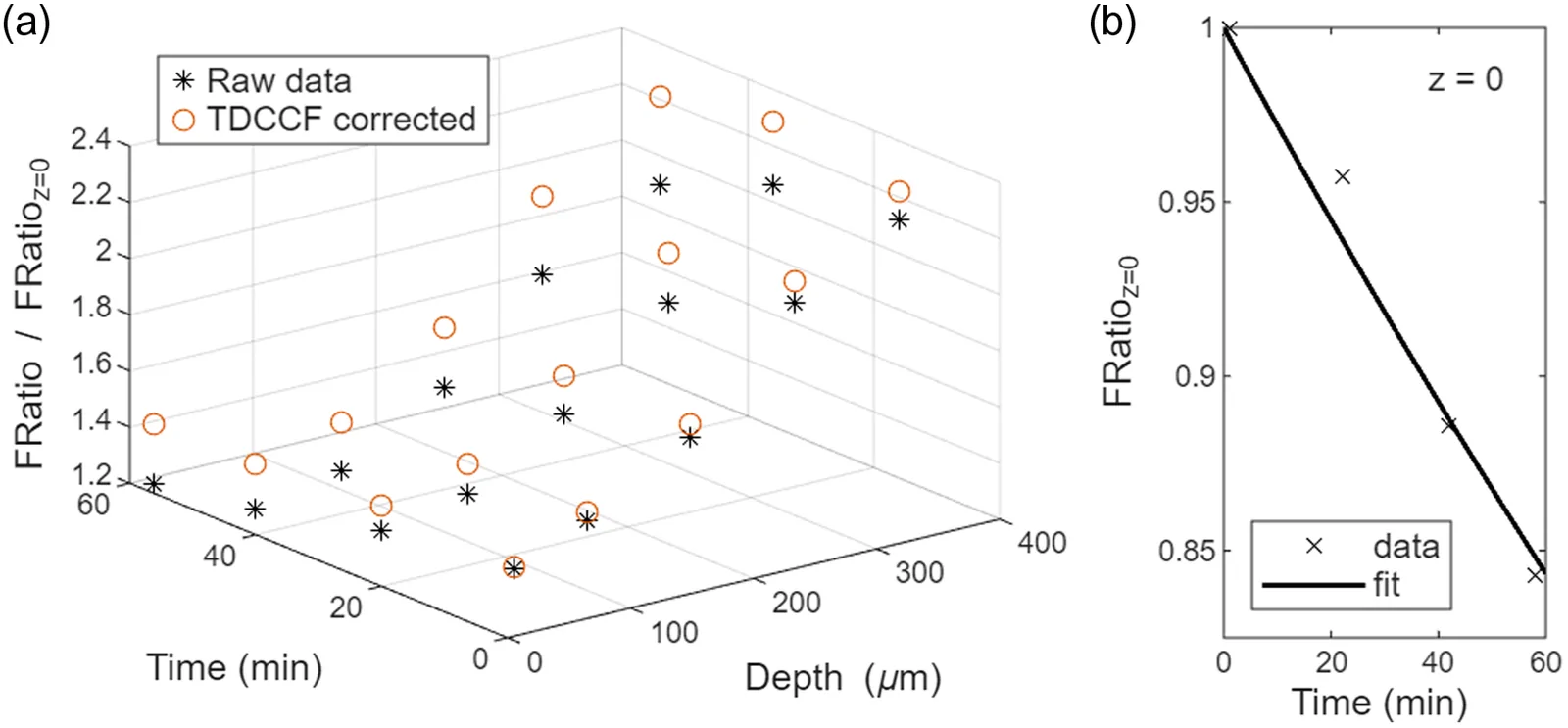
Determination of the kinetics of the differential change in concentration of the fluorophores in the blood. (a) Three consecutive repetitions of the fluorescence ratio measurement at different depths were performed. Raw data: FRatio(z,t)/FRatio(0,0). TDCCF corrected (Sec. 2.3): FRatio(z,t)/FRatio M(0,t). (b) Experimental measurements at z=0 were fitted by an exponential to get FRatio M(0,t).

As explained in Sec. 2.3, $\mathrm{FRatio}(z_{i},t_{i})$ needs to be normalized by the fluorescence ratio at the surface and at the same time, FRatio (0, $t_{i}$) to isolate depth-dependent effects [see Fig. 11(a)]. Since measurements at depth and at the surface were not acquired simultaneously, we first modelled the time-dependence of changes in the concentration of fluorophores (TDCCF) at the surface in order to estimate the fluorescence ratio at the surface FRatio M(0,t) at any time [Fig. 11(b)].

## 7 Appendix B: Reformulation of Eqs. 15-16 and Eqs. 18-20 using $a_{e}$

When the extinction coefficient $\mu_{e}(\lambda )$ is expressed as the product of $\mu_{t}^{\prime }(\lambda )$ by the coefficient of proportionality $a_{e}(\lambda )$, Eqs. (15) and (16) become (Sec. 3.3.2)

$$F(\lambda )=\frac{-2a_{e}(\lambda {)}^{2}}{a_{e}(\lambda {)}^{2}-\frac{3\mu_{a}(\lambda )}{\mu_{t}^{\prime }(\lambda )}}$$ (27)

$$G(\lambda )=\frac{3(a_{e}(\lambda {)}^{2}\mu_{t}^{\prime }(\lambda )-\mu_{a}(\lambda ))}{a_{e}(\lambda {)}^{2}\mu_{t}^{\prime }(\lambda )-3\mu_{a}(\lambda )}+\frac{3a_{l}}{\mu_{t}^{\prime }(\lambda )+a_{l}k_{d}(\lambda )}(\mu_{t}^{\prime }\left(\lambda \right)-\frac{a_{e}(\lambda )k_{d}(\lambda )+\mu_{a}(\lambda )}{a_{e}(\lambda )+\sqrt{\frac{3\mu_{a}(\lambda )}{\mu_{t}^{\prime }(\lambda )}}}).$$ (28)

And Eqs. (19)–(21) become

$${\left(\Phi_{NA34}(z)\right)}_{AbSc}=\int_{\lambda 3}^{\lambda 4}\alpha (\lambda )\varphi_{NA}(\lambda )(F(\lambda )e^{-a_{e}(\lambda )\mu_{t}^{\prime }(\lambda )z}+G(\lambda )e^{-k_{d}(\lambda )z})d\lambda$$ (29)

$${\left(\Phi_{NB34}(z)\right)}_{AbSc}=\int_{\lambda 3}^{\lambda 4}\alpha (\lambda )\varphi_{NB}(\lambda )(F(\lambda )e^{-a_{e}(\lambda )\mu_{t}^{\prime }\left(\lambda \right)z}+G\left(\lambda \right)e^{-k_{d}\left(\lambda \right)z})d\lambda$$ (30)

$${\left(\Phi_{NB12}(z)\right)}_{AbSc}=\int_{\lambda 1}^{\lambda 2}\alpha (\lambda )\varphi_{NB}(\lambda )(F\left(\lambda \right)e^{-a_{e}\left(\lambda \right)\mu_{t}^{\prime }(\lambda )z}+G(\lambda )e^{-k_{d}(\lambda )z})d\lambda .$$ (31)

## 8 Appendix C: Derivation of Equation 25

Derivation of equations from Sec. 3.3.4: $K_{A}=\frac{\left[S][M\right]}{\left[MS\right]}$, which can be rewritten $[S]=\frac{K_{A}[MS]}{\left[M\right]}$.

As the total concentration of bound and free sensor is $c_{S}=[MS]+[S]=[MS]+\frac{K_{A}[MS]}{\left[M\right]}$, therefore $[MS]=\frac{c_{S}}{\left(1+K_{A}/[M]\right)}$.

This can be rewritten: $[M]=\frac{K_{A}}{c_{S}/[MS]-1}$ and gives Eq. (25).

## 9 Appendix D: Calculation of pH in different Conditions

Here are the expressions used in Sec. 3.3.4 to calculate M(z) in different conditions. Calculation of concentration of M from raw data is given by

$$[M](z)=\frac{K_{A}}{\frac{c_{S}\sigma_{N}\Phi_{N0MS34}}{\left[B](\frac{N_{0\lambda 3\lambda 4}}{N_{0\lambda 1\lambda 2}}\left([MS],[B]\right)\Phi_{N0B12}-\Phi_{N0B34}\right)}-1}.$$ (32)

Calculation of concentration of M with exponential fit correction is given by

$$[M](z)=\frac{K_{A}}{\frac{c_{S}\sigma_{N}\Phi_{N0MS34}}{\left[B]({\left(\frac{N_{\lambda 3\lambda 4}}{N_{\lambda 1\lambda 2}}\right)}_{\mathrm{ExpFit}}([MS],[B])(z)e^{-uz}\Phi_{N0B12}-\Phi_{N0B34}\right)}-1}.$$ (33)

TDCCF correction compensates for eventual change in the $\frac{c_{S}}{\left[B\right]}$ with time and this ratio can thus be considered as constant.

## 10 Appendix E: Effect of Edges in Fluorescence Ratio in Venules

**Fig. 12:**
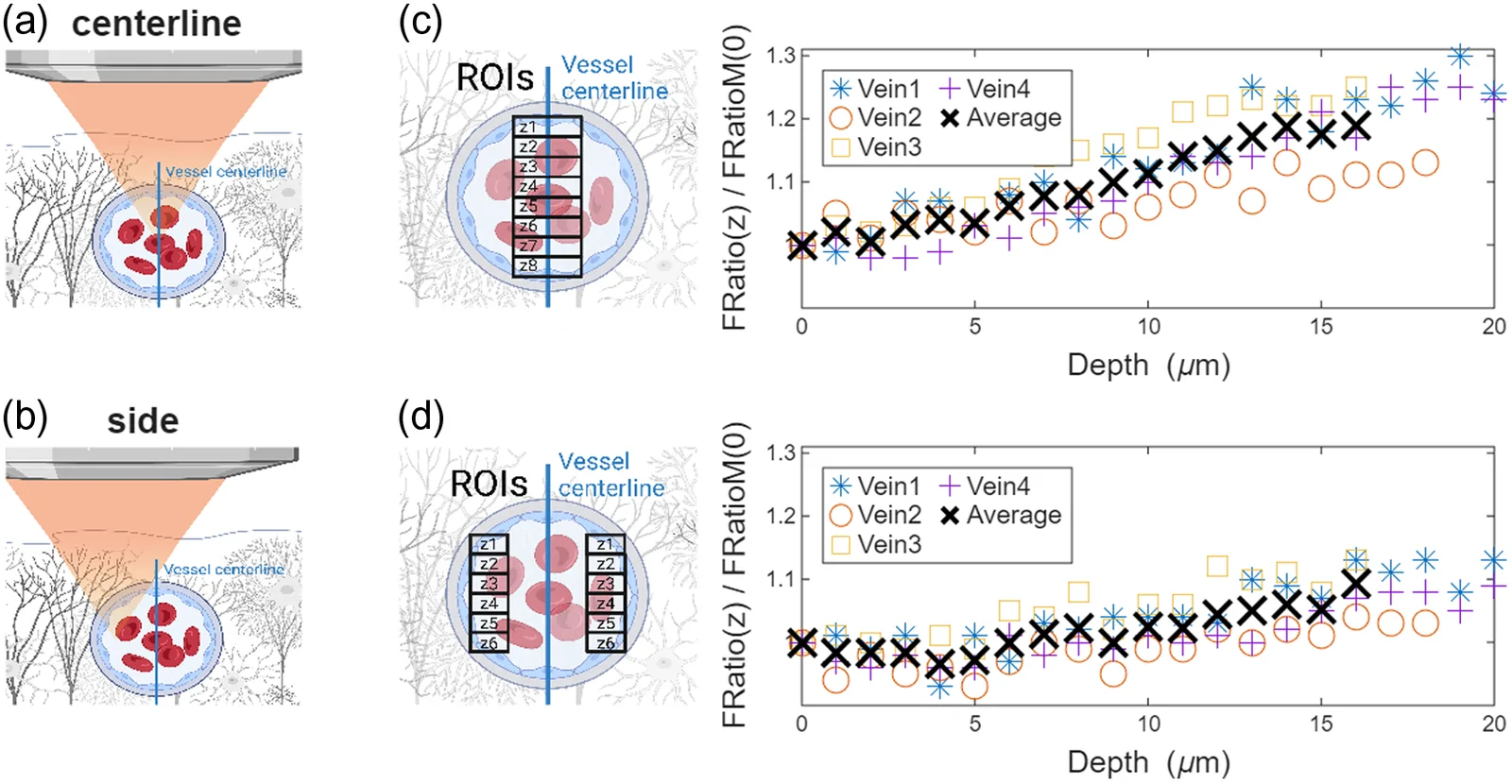
Effect of edges on fluorescence ratio in venules. (a) Scheme showing the cone of emitted photons when the focus is on the vessel axis. (b) Same as (a) for a focus away from the optical axis. (c) Scheme showing ROIs centered on the optical axis used to data analysis from 4 venules and resulting FRatio(z)/FRatioM(0) versus depth. (d) Same as (c) for ROIs away from the optical axis (Sec. 3.3.4).

As mentioned in Sec. 3.3.3, fluorescence photons emitted along the vessel centerline traverse similar path lengths within the vessel cross-section [Fig. 12(a)] whereas fluorescence photons emitted off-axis experience de much wider range of path lengths before collection [Fig. 12(b)]. Consequently, the depth-dependance of the red-to-green fluorescence ratio is expected to be sensitive to the choice of the ROI. To test this hypothesis, we compared ROIs centered on the vessel centerline with ROIs positioned away from the centerline [Figs. 12(c), 12(d)] in a set of venules.

## 11 Appendix F: Practical Guide

### 11.1 Establishment of the Depth-Dependent Correction in the Brain Region of Choice

Step 1: Evaluate if the relative concentration of fluorophores might change with time in control conditions (TDCCF).
  If yes, measure the fluorescence ratio versus time in control conditions at the surface to get the correction for these time-dependent changes. (Matlab script from Appendix A Fig. 11).
Step 2: Measure the depth-dependence in the brain region of interest.
  Decide how many layers should be considered and adjust the necessary sampling for each layer.
  Measurement of the TDCCF and depth dependence can be combined as in Appendix A.
Step 3: Calculate the values for $a_{e}$ and $\chi_{\mathrm{Blood}\;\mathrm{CC}}$ using data collected in steps 1 and 2 and Eq. (17) (Matlab script from Fig. 5).

### 11.2 Correction of the Depth-Dependence in the Brain Region of Choice Using Known Parameters of the AbSc Model

Step 1: Establish the equation between the biomarker M and the concentration of the fluorescent molecule MS.
Step 2: Combine equations from Sec. 11.2 Step 1 to Eq. (17) to measure biomarker M. Use either the $a_{e}$ and $\chi_{\mathrm{Blood}\;\mathrm{CC}}$ values calculated in Sec. 11.1 or reference values.

## Data Availability

The data presented in this article and developed code are publicly available in GitHub at https://github.com/laboratory-charpak/Multiphoton-Ratiometric-Measurements-Model.
